# Light-activated gold nanorod vesicles with NIR-II fluorescence and photoacoustic imaging performances for cancer theranostics

**DOI:** 10.7150/thno.44376

**Published:** 2020-03-26

**Authors:** Xiaoguang Ge, Qinrui Fu, Lichao Su, Zhi Li, Wenmin Zhang, Tao Chen, Huanghao Yang, Jibin Song

**Affiliations:** MOE key Laboratory for Analytical Science of Food Safety and Biology, College of Chemistry, Fuzhou University, Fuzhou 350108, China

**Keywords:** metallodrugs, second near infrared window, photoacoustic imaging, fluorescence imaging, gold nanorod vesicle

## Abstract

Fluorescence (FL) and photoacoustic (PA) imaging in the second near infrared window (NIR-II FL and NIR-II PA) hold great promise for biomedical applications because of their non-invasive nature and excellent spatial resolution properties.

**Methods**: We develop a NIR-II PA and NIR-II FL dual-mode imaging gold nanorod vesicles (AuNR Ves) by self-assembly of amphiphilic AuNR coated with light responsive polyprodrug of Ru-complex and PEG, and NIR-II cyanine dye (IR 1061). The AuNR Ves showed strong ligh absorption property and PA imaging performance in the NIR-II windows. Moreover, the NIR-II fluorescence signal of IR 1061 loaded in the AuNR Ve is quenched.

**Results**: The AuNR Ves can release photosensitizer Ru-complex and IR 1061 sequentially triggered by NIR light irradiation, leading to a corresponding NIR-II PA signal decrease and NIR-II FL signal recovery. Meanwhile, Ru-complex can not only serve as a chemotherapeutic drug but also generate singlet oxygen (^1^O_2_) under NIR light irradiation. The release of Ru-complex and photodynamic therapy are guided by the responsive variation of NIR-II PA and NIR-II FL signals.

**Conclusions**: The AuNR Ve possessing not only precisely control ^1^O_2_/drug release but also the intrinsic ability to monitor therapy process offers a new strategy for the development of smart theranostic nanoplatform.

## Introduction

Metal-drugs have been greatly developed in cancer treatment since the success of several FDA (US Food and Drug Administration)-approved platinum anticancer drugs 1. Nowadays, various metal complexes, such as cis-platinum, carboplatin, oxaliplatin, and imidazolium trans[tetrachloro-(dimethylsulfoxide)imidazole ruthenium(III)] (NAMI-A) have entered clinical trial stage 2-5. Among all these metal complexes, Ru-complex is a promising anticancer metallodrug that has been widely used for photodynamic therapy (PDT) and DNA detection owing to its excellent photophysical properties 4, 6-9. However, the development of anticancer Ru-complex has been greatly limited because it lacks of selectivity between tumor and healthy cells 10, 11. Moreover, Ru-complex, being a photosensitizer for PDT, emits low-efficiency ROS.

Photoactivation is an elegant method that can convert nontoxic prodrugs to active cytotoxic drugs in a spatially and temporally controlled manner. Thus, the side effects of anticancer drugs have been greatly reduced 3, 12-15. Because of the success of transition metal complexes in photoinduced ligand release with low-energy light, ruthenium (II)-based systems have been developed for caging in a manner that is inherently different from traditional organic chromophores 3, 16-19. More importantly, these complexes can be photoactivated at the tumor site to release bioactive Ru species or ligands. The Ru-complex remains inert until it is triggered by low-density light, which induces ligand release, because of lowest-lying triplet metal-to-ligand charge transfer (^3^MLCT) states 6, 20. Moreover, Ru-complex coordinated with polypyridine can produce singlet oxygen (^1^O_2_) under laser irradiation 21. Therefore, Ru-complex has excellent performance for against cancer compared with cisplatin owing to the combination of PDT and chemotherapy 22-24. Nevertheless, Ru-complex has a poor biocompatibility and low tumor accumulation efficiency because of its positive charge and small size with a short duration of circulation in the blood 10. Consequently, the abovementioned problems limit the application of Ru-complex *in vivo*. To overcome these shortcomings, Ru-complex was introduced into a polymer, which can self-assemble into nanoparticles to prolong its duration of blood circulation and efficiently accumulate in tumor 25. However, Ru-complex was usually activated by visible light, or by the conversion of upconverting nanoparticles from near infrared region (NIR) to visible light. It is generally known that NIR light can penetrate biological tissue compared with visible light 26. Therefore, the development of NIR light-activatable nanoplatform is necessary and urgent.

With the development of optical technology, light-induced imaging and therapy have attracted wide attention for cancer theranostics 27, 28. So far, photoacoustic (PA) and fluorescence (FL) imaging have seen considerable expansion within the past few years 29-38. However, previous PA and FL imaging were mainly in the first NIR window (650 nm-900 nm) with low penetration and high background signals, limiting their *in vivo* application. Recently, many studies have revealed that PA and FL imaging in the second near infrared window (1000-1700 nm) (NIR-II) showed non-invasive and excellent spatial resolution for tumor diagnosis 39, 40. Compared with conventional optical imaging in the first NIR window, NIR-II FL and NIR-II PA imaging were superior for biological imaging because of its higher signal-to-noise ratio, better imaging qualities, and deeper tissue penetration 41. NIR-II FL imaging enables direct and wide visualization of dynamic biological tissues of interests, including cells *in vitro* and organs or tissues *in vivo,* with high spatio-temporal resolution and sensitivity 42. In contrast, NIR-II PA imaging has the capability to delineate physiological metabolism and anatomic structure with a microscopic spatial resolution given its combination of optical and ultrasound imaging 43. However, it is difficult to obtain further comprehensive and accurate diagnosis information by only a single imaging modality. Thus, dual-mode optical imaging with NIR-II FL imaging and PA imaging has the potential to improve diagnostic accuracy. The responsive dual NIR-II PA and NIR-II FL imaging-guided synergistic chemo-PDT nanomaterials has rarely been reported.

Because of the presence of tunable localized surface plasmon resonance (LSPR), photophysical properties, and biocompatibility, gold nanorods (AuNR) not only serve as imaging probes for PA imaging but also act as a drug-loading platform 44. Moreover, plasmonic coupling between gold nanocrystals generates an enhanced electromagnetic (EM) field, leading to an enhanced light absorption efficiency, thus providing a stronger PA signal 45. Furthermore, the strong light absorption of the AuNR assembly can quench fluorescence signal of the fluorescence dye as the excitation or emission light of the fluorescent molecules is absorbed.

**Figure 1:**
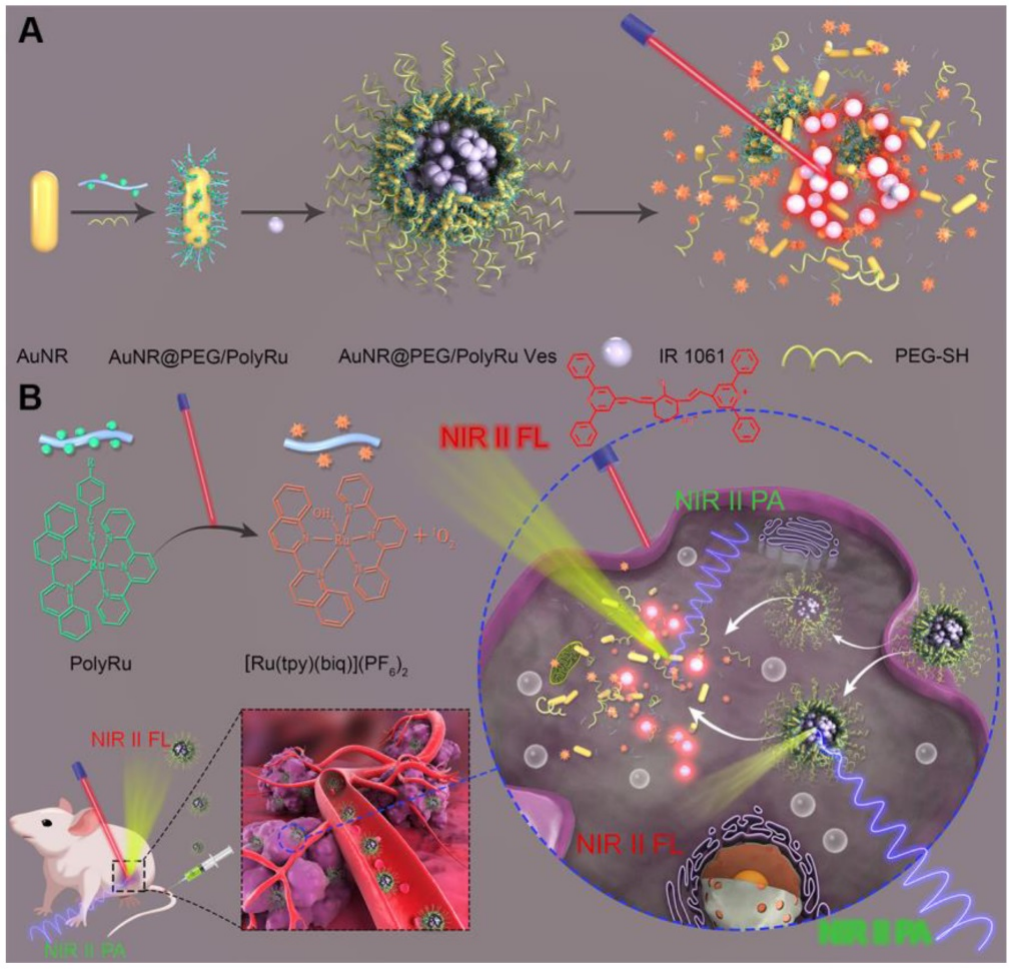
** (A)** Schematic illustration of the preparation a NIR light-activatable AuNR@PEG/PolyRu Ves by self assembly of AuNR coated with PEG and light responsive polyprodrug PolyRu, and NIR-II fluorescence dye (IR1061). **(B)** *In vivo* accumulation of the AuNR@PEG/PolyRu Ves and disassociation of the nanoplatform after NIR laser irradiation, leading to sequential generation of ^1^O_2_ and release of chemotherapy drug Ru complex. Dual-responsive NIR-II fluorescence and photoacoustic imaging was employed to guid cancer synergistic chemo-PDT cancer therapy.

In this study, we first developed a dual NIR-II PA and FL imaging responsive AuNR vesicle nanoplatform by self-assembly of amphiphilc AuNR coated with light-responsive polyprodrug of Ru-complex (PolyRu) and poly(ethylene glycol) (PEG), and NIR-II IR 1061 dye **(Figure 1A)**. This nanoplatform can release photosensitizer Ru-complex and IR 1061 sequentially on being triggered by NIR light, leading to the corresponding variation of NIR-II PA and FL signals **(Figure 1B)**. Ru-complex was introduced into the side chain of the polymer (PolyRu). Previously, PolyRu could only release Ru-complexes again after being triggered by visible light *via* ^3^MLCT, limiting its *in vivo* application. PEG can prolong the duration of circulation of these nanoparticles in the blood and reduce nonspecific cellular uptake. Given the strong plasmonic coupling effect between AuNRs in the vesicular shell, the vesicle showed enhanced light absorption efficiency in the NIR-II window. Benefitting from the strong EM field generated by AuNR vesicle, the light absorption property of the PolyRu grafted on the surface of AuNR also increased. Moreover, PolyRu could be disintegrated under NIR light irradiation to release Ru-complex. In addition, NIR-II FL signal of the loaded IR 1061 was quenched, because its FL spectrum peak matched well with the LSPR peak of the AuNR vesicle. When the AuNR Ve was disassembled by NIR laser irradiation, NIR-II FL signal was recovered and NIR-II PA signal decreased at the same time. Meanwhile, the Ru-complex acts as a chemotherapy drug and photosensitizer, which has a chemotherapeutic and PDT effect on cancer. Thus, light-responsive NIR-II PA and FL imaging can be used to guide the synergistic cancer PDT and chemotherapy with high efficiency. The AuNR Ves have excellent biocompatibility and satisfactory therapeutic effect *in vitro* and *in vivo*.

## Results and Discussion

### Preparation of NIR Light-Responsive Polymer Nanoparticles

To prepare light-responsive AuNR vesicle, NIR light-activated Ru-containing polymer of PolyRu was first synthesized and characterized **(Figures S1-S4)**. The ruthenium complex [Ru(tpy)(biq)](PF_6_)_2_ was prepared *via* the coordination of Ru(III) with 2,2′:6′,2′′-terpyridine (tpy) and 2,2′ biquinoline (biq). Subsequently, poly(methacrylic acid) (PAA) was synthesized by polymerization of tertiary-butyl methacrylate through the atom transfer radical polymerization (ATRP) method. After polymerization, the protective tert-butyl group was removed. The poly(6-(4-cyanophenoxy) hexyl methacrylate) (PCPH)-containing cyano group in the side chains was obtained by the ATRP method and covalent grafting of 6-(4-cyanophenoxy) hexyl (CPH). Finally, a photoactivatable Ru-containing polymer (PolyRu) was constructed *via* coordination between the cyano groups of PCPH and [Ru(tpy)(biq)](PF_6_)_2_.

**Figure 2:**
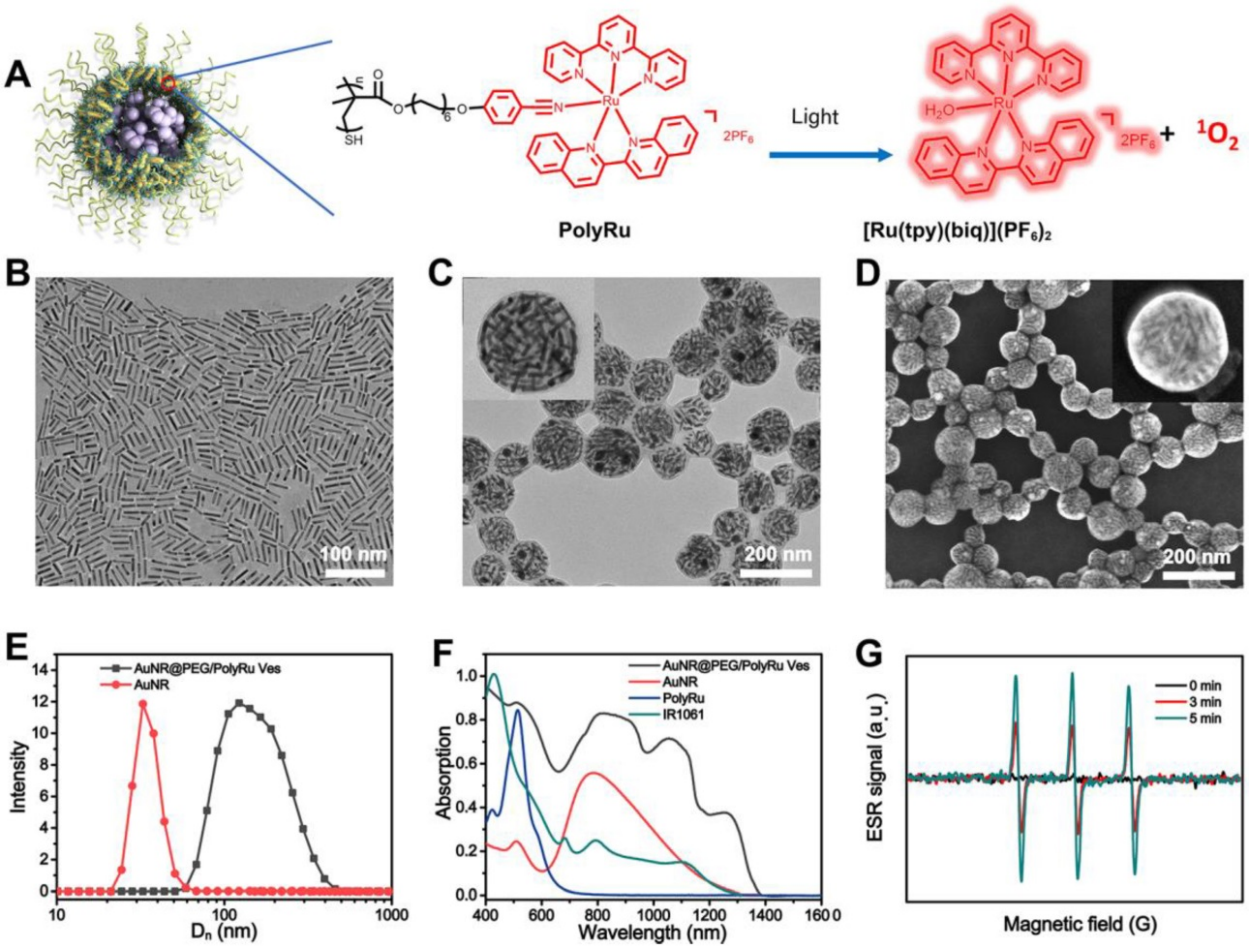
** (A)** Scheme of self-assembly of AuNR coated with PEG and PolyRu into AuNR Ve, and generation of ^1^O_2_ and release Ru complex triggered by NIR laser irradiation. TEM images of AuNR **(B)**, and AuNR@PEG/PolyRu Ves** (C)**. **(D)** SEM images of AuNR@PEG/PolyRu Ves. **(E)** Hydrodynamic distribution of the AuNR@PEG/PolyRu Ves and AuNR. **(F)** UV-*vis* spectra of PolyRu, AuNR, IR1061, and AuNR@PEG/PolyRu Ves. **(G)** The generation of ^1^O_2_ was identified by ESR spectroscopy.

AuNR with the length of 35 nm and width of 7 nm was first prepared. Dual NIR-II PA and FL responsive AuNR@PEG/PolyRu Ves were prepared *via* a self-assembly approach that has been previously reported **(Figure 2A)**. In brief, AuNR@PEG/PolyRu and IR 1061 were first dispersed into chloroform. Then, a certain proportion chloroform and polyvinyl alcohol (PVA) aqueous solution (3 mg mL^-1^) were mixed. Following this, the mixed solution was emulsified by ultrasonic emulsification. This emulsion was evaporated at room temperature to remove the chloroform phase. As shown in transmission electron microscopy (TEM) images, the AuNRs were closely attached with each other and formed a vesicular shell **(Figure 2B, 2C)**. Then, the element mapping reults of Au and Ru further supported that AuNR@PEG/ PolyRu was successfully prepared** (Figure S5)**. Moreover, scanning electron microscopy (SEM) images of the AuNR@PEG/PolyRu Ves demonstrated the vesicle with well spherical nanostructures **(Figure 2D)**. Dynamic light scattering (DLS) revealed that the average size of the AuNR@PEG/PolyRu Ves was approximately 95 nm **(Figure 2E)**. Ru-complex-loading efficiency was calculated by determining the change in Ru-complex before and after coordination with the polymer PCPH through a standard curve of Ru-complex **(Figure S6)**. The Ru-complex- loading content proportion of the AuNR@PEG/PolyRu Ves was ~10.1% for the following experiments involving Ru-complex release and *in vitro* and *in vivo* treatments.

Furthermore, UV-vis spectra was used to characterize the AuNR@PEG/PolyRu Ves. As shown in **Figure 2F**, the characteristic peak of PolyRu at 510 nm was observed in the UV-vis spectra of AuNR@PEG/ PolyRu Ves, indicating that PolyRu had been successfully conjugated on the AuNR surface. The absorption intensity of PolyRu in NIR I was enhanced because of the EM field generated by AuNR. This result exhibited favorable property for efficient optical interactions between of them. The characteristic peak of AuNR was observed at 800 nm. However, light absorption of AuNR was not observed in the NIR-II window. In contrast to AuNR, a significant absorption peak redshift of AuNR@PEG/ PolyRu Ves was observed in the NIR-II window because of the plasmonic coupling between AuNRs in the vesicular shell.

To further demonstrate the generation ability of ^1^O_2_ under 808 nm NIR laser irradiation, the electron spin resonance (ESR) technique was used to detect ^1^O_2_ using 2,2,6,6-tetramethylpiperide (TEMP) as a spin-trapping adduct. The ESR spectra was observed to be dependent on the irradiation time, indicating the generation of ^1^O_2_ by AuNR@PEG/PolyRu Ves under NIR laser irradiation **(Figure 2G)**. As a control group, the ESR spectra of AuNRs under 808 nm NIR laser irradiation showed a ignored generation of ^1^O_2_ **(Figure S7)**.

### NIR Light-Triggered Dis-assembly of the AuNR@PEG/PolyRu Ves and Drug Release

**Figure 3:**
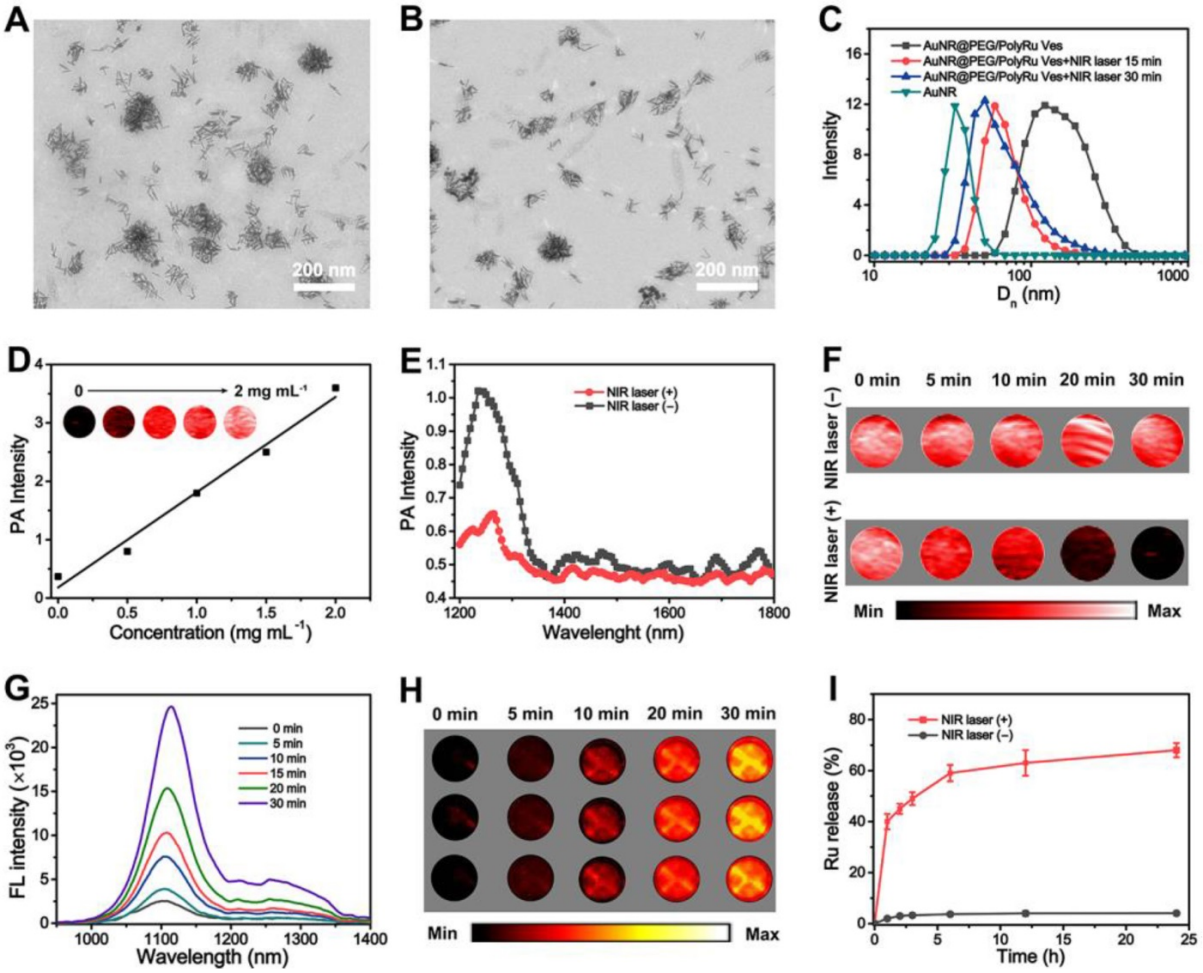
TEM images of AuNR@PEG/PolyRu Ves after NIR laser irradiation (50 mW cm^-2^) for **(A)** 15 min and **(B)** 30 min, and **(C)** Hydrodynamic distribution of the AuNR@PEG/PolyRu Ves before and after NIR laser irradiation. **(D)** NIR-II PA images of AuNR@PEG/PolyRu Ves at different concentration. **(E)** PA spectra of the AuNR@PEG/PolyRu Ves before and after NIR laser irradiation (50 mW cm^-2^) for 5 min. **(F)** PA images of AuNR@PEG/PolyRu Ves treated with or without NIR laser irradiation (50 mW cm^-2^). **(G)** FL spectra of AuNR@PEG/PolyRu Ves treated 808 nm laser irradiation (1 W cm^-2^) for different times. **(H)** NIR-II FL images of the vesicle solution under NIR laser irradiation (180 mW cm^-2^) for different times (three parallet groups). **(I)** The Ru-complex release profile of the AuNR@PEG/PolyRu Ves treated with or without 808 nm laser irradiation (50 mW cm^-2^).

We further investigated the NIR light activation of Ru-complexes in the side chain of PolyRu. The release of Ru-complexes from PolyRu after laser irradiation could be detected by exposed time-dependent UV-vis absorption spectroscopy. We recorded the UV-vis absorption changes of PolyRu (acetone/H_2_O 1:1) under NIR laser irradiation (660 nm, 50 mW cm^-2^). As shown in **Figure S8**, a decrease of absorption at 510 nm and an increase in the peak at 560 nm under a 660-nm laser irradiation. The Ru-complex was nearly completely released within 30 min. However, there were no obvious changes at 510 nm with NIR laser irradiation (808 nm, 50 mW cm^-2^) (**Figure S9**). These results suggested that PolyRu is light activatable. As shown in TEM images, the morphology of the AuNR@PEG/PolyRu vesicles was also gradually disrupted into small clusters **(Figure 3A, Figure S10)** and single AuNRs **(Figure 3B)** after NIR laser irradiation after 2 min, 5 min, 15 min and 30 min, respectively. The fast disassembly of the AuNR@PEG/PolyRu Ves triggered by NIR laser irradiation was further verified by a size decrease in the DLS data **(Figure 3C)**. The PolyRu shell was removed after the nanoparticle was treated with NIR laser irradiation. The reason for this is associated with the splitting of PolyRu and the disassembly of the polymeric shell.

Given the NIR light absorption enhancement effect of the assembled AuNR, a PA signal redshift to the NIR-II windows was observed. PA images of the AuNR@PEG/PolyRu Ves and AuNR dispersed in water with different concentrations were detected by NIR-II laser irradiation (1240 nm)** (Figure 3D, S11)**. PA images of the AuNR@PEG/PolyRu Ves were brighter than those of AuNR. In addition, quantitative analysis demonstrated that PA signals of AuNR@PEG/PolyRu Ves were stronger than those of AuNR, which nearly displayed no PA signal on being exposed to 1240 nm laser irradiation. Moreover, PA signals of AuNR@PEG/PolyRu Ves were dependent on the concentration. To explore the changes in PA signals, PA spectrum of the AuNR@PEG/PolyRu Ves dispersed in water was recorded. The peak of PA spectra was at 1240 nm belong to AuNR@PEG/ PolyRu Ves, which was consistent with its absorption spectrum **(Figure 3E)**. However, when the aqueous solution of AuNR@PEG/PolyRu Ves was exposed to laser irradiation of 808 nm (50 mW cm^-2^) for 5 min, a blue-shift in the absorption peak and a significant decrease on the intensity were observed. To study the response of NIR light with time, PA images of AuNR@PEG/PolyRu Ves were observed in an Eppendorf tube with or without NIR laser irradiation. When AuNR@PEG/PolyRu Ves aqueous solution was irradiated under 808 nm laser, gradual decrease of PA signal was detected** (Figure 3F)**. However, PA signal of AuNR@PEG/PolyRu Ves remained unchanged without NIR laser irradiation.

NIR-II FL signal of IR1061 in the AuNR@PEG/PolyRu Ves solution was very weak because of the fluorescence quenching effect of AuNR Ves **(Figure S12)**. NIR-II FL signals of IR 1061 in the AuNR@PEG/PolyRu Ves were mostly increased with NIR laser irradiation **(Figure 3G)**. This was because after treatment with 808 nm laser (50 mW cm^-2^), the IR 1061 was released from AuNR@PEG/PolyRu Ves and its fluorescence signal was recovered. In contrast, there was no obvious change in the fluorescence signals of IR1061 without NIR laser irradiation. NIR-II FL images were detected *in vitro* by FL imaging using 96-well plates with or without NIR laser irradiation. Negligible fluorescence signals were observed from FL imaging *in vitro* without NIR laser irradiation **(Figure S13)**. In contrast, a strong fluorescence signal was detected with NIR light irradiation **(Figure 3H)**. The fluorescence signal was observed to increase with increasing exposure time of NIR laser irradiation. This result was agreeing with the abovementioned results of the responsive variation in the fluorescence spectrum. Therefore, dual NIR-II FL and PA signals were changed resulting from the disassembly of AuNR@PEG/PolyRu Ves, which could be used to monitor and guide their *in vivo* behavior.

To benefit from the NIR laser responsive disassociation of vesicles, we further investigated the release of Ru-complexes triggered by NIR laser irradiation *in vitro*. More than 50% Ru-complexes were released from the AuNR@PEG/PolyRu Ves rapidly in 5 h after NIR laser treatment for 5 min. However, negligible Ru-complexes released without NIR laser treatment **(Figure 3I)**, indicating its stability in a physiological environment. AuNR@PEG/PolyRu Ves emerged to have favorable photo lability as the polyprodrug of Ru-complexes was an NIR light-activatable polymer. Hence, NIR-II FL and PA imaging can be used to monitor the disassembly of AuNR@PEG/PolyRu Ves and the release of Ru-complex.

### *In Vitro* Cytotoxicity and Imaging of NIR Light-Responsive AuNR@PEG/PolyRu Ves

**Figure 4:**
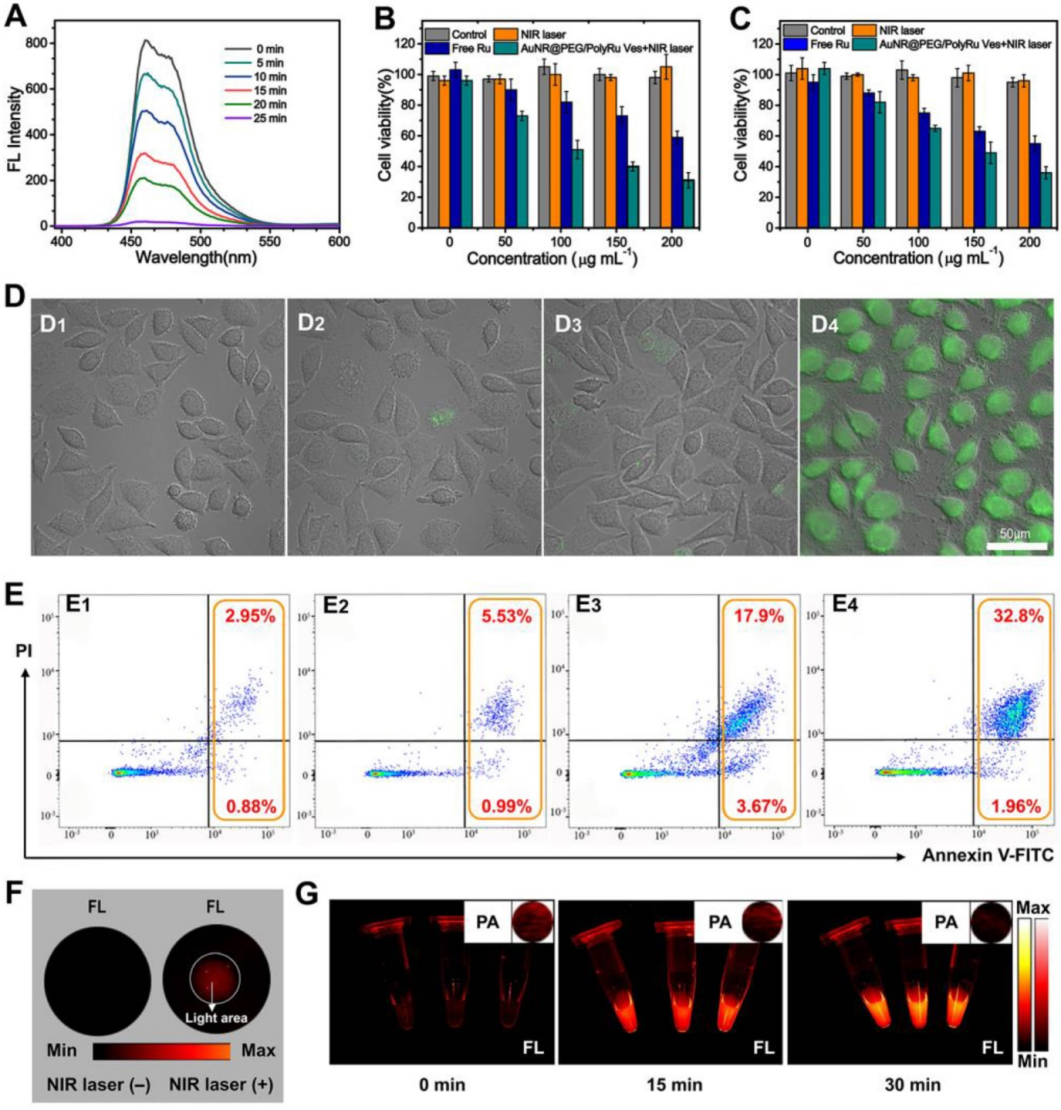
** (A)** Fluorescence spectra of DPBF treated with AuNR@PEG/PolyRu Ves and NIR laser irradiation. Cell viability of **(B)** MCF-7 and **(C)** 4T1 cells treated with the samples with or without laser irradiation (50 mW cm^-2^, 5 min). **(D)** DCFH fluorescence of MCF-7 cells exposed to PBS buffer (D1), NIR laser irradiation (D2), AuNR@PEG/PolyRu Ves without NIR laser irradiation (D3), AuNR@PEG/PolyRu Ves after NIR laser irradiation (D4). Scale bars: 50 µm. **(E)** Cell apoptosis analysis of MCF-7 cells by flow cytometer after incubation with PBS (E1), AuNR@PEG/PolyRu Ves without NIR laser irradiation (E2), free Ru (E3), and AuNR@PEG/PolyRu Ves with NIR laser irradiation (E4).** (F)** NIR-II FL image of the MCF-7 cells treated with AuNR@PEG/PolyRu Ves before and after NIR laser irradiation. (G) NIR-II FL and NIR-II PA images (inset) of the concentrated suspending solution of MCF-7 cells in PBS after NIR laser irradiation for different times.

Chemotherapy and PDT are both effective cancer therapy approaches. For the vesicle, the released ^1^O_2_ and [Ru(tpy)(biq)(H_2_O)]^2+^ from the vesicle triggered by NIR laser are anticancer materials for PDT and chemotherapy, respectively. While studying the generation of ^1^O_2_ by 808 nm laser irradiation, 1, 3-diphenylisobenzofuran (DPBF) was used as a fluorescence probe for the detection of ^1^O_2_. The fluorescence signal of DPBF gradually reduced because ^1^O_2_ can break the structure of DPBF **(Figure 4A)**. With an increase in exposure time, the production efficiency of ^1^O_2_ by AuNR@PEG/PolyRu Ves under NIR laser irradiation increased gradually. Following this, the CCK-8 assay was used to evaluate the cytotoxicity of AuNR@PEG/PolyRu Ves against MCF-7 cells and 4T1 cells. After NIR laser irradiation (808 nm, 50 mW cm^-2^), the cell viability (MCF-7 cells: 31±5%, 4T1 cells: 36±3%) for AuNR@PEG/PolyRu Ves was found to be lower than that of free Ru-complexes (MCF-7 cells: 59±4%, 4T1 cells: 55±5%) and AuNR@PEG/PolyRu Ves without NIR laser irradiation (MCF-7 cells: 105±5%, 4T1 cells: 96±4%)** (Figure 4B, 4C, S14A)**. Compared with the cell viability of free Ru-complexes, there was negligibl changes of cell viability for Ru-complexes under NIR laser irradiation** (Figure S14B)**. These results suggested that AuNR@PEG/PolyRu Ves emerged to have excellent biocompatibility without NIR laser irradiation and showed significant cytotoxicity under NIR laser irradiation. Comparing with the results in dark condition, NIR laser irradiation could convert nontoxic prodrugs to active cytotoxic drugs. To detect the intracellular photosensitized^ 1^O_2_, DCFH-DA was used as a fluorescent probe **(Figure 4D)**. Subsequently, the cells were observed by confocal laser scanning microscopy (CLSM). Infinitesimal green fluorescence was observed in the AuNR@PEG/ PolyRu Ves without NIR laser irradiation group, free Ru group, and PBS group. In contrast to the abovementioned three groups, an intense green fluorescence signal was detected in the presence of AuNR@PEG/PolyRu Ves with NIR laser irradiation, indicating the efficient generation of intracellular ^1^O_2_.

Additionally, flow cytometry analysis was used to evaluate cellular apoptosis by staining MCF-7 cells with annexin V FITC/propidium iodide (PI) after treatment with PBS, free Ru, AuNR@PEG/PolyRu Ves with and without NIR laser irradiation. As shown in **Figure 4E**, PBS and AuNR@PEG/PolyRu Ves without NIR laser irradiation induced the apoptotic cell levels of 3.83% and 6.52%, respectively. The result revealed that AuNR@PEG/PolyRu Ves showed excellent biocompatibility and low dark toxicity. However, the apoptotic cell levels of free Ru and AuNR@PEG/PolyRu Ves with NIR laser irradiation were modestly increased to 21.57% and 34.76%, respectively, resulting from chemotherapy against free Ru and a combination of chemotherapy and PDT, respectively. Briefly, flow cytometry analysis confirmed that the synergistic effect of AuNR@PEG/PolyRu Ves treated with NIR laser irradiation can induce significantly enhanced cancer cell killing efficacy because of the synergistic effect of PDT and chemotherapy inside the cells.

To study the intracellular response of NIR-II fluorescence, MCF-7 cells were first inoculated with AuNR@PEG/PolyRu Ves for 12 h on a 6-well plate **(Figure 4F)**. Fluorescence signal was observed in the region irradiated with NIR laser because the dissociation of AuNR@PEG/PolyRu Ves triggered NIR-II fluorescence recovery of IR 1061. Therefore, AuNR@PEG/PolyRu Ves could be activated by NIR laser and showed limited side effects in the dark region. Meanwhile, NIR-II PA and FL signals were observed in a plastic tube **(Figure 4G)**. NIR-II PA and FL signal showed a gradual weakening and increasing trend, respectively, after NIR laser irradiation, which were consistent with the variation of NIR-II FL and PA intensities of AuNR@PEG/ PolyRu Ves under NIR laser irradiation. These results suggested that NIR laser-activatable AuNR@PEG/ PolyRu Ves promoted chemotherapy and PDT effects to induce cell apoptosis. In addition, the changes in NIR-II PA and FL signals could guide chemotherapy and PDT *in vivo*.

### *In Vivo* NIR Light-Responsive NIR-II PA and FL Imaging

**Figure 5:**
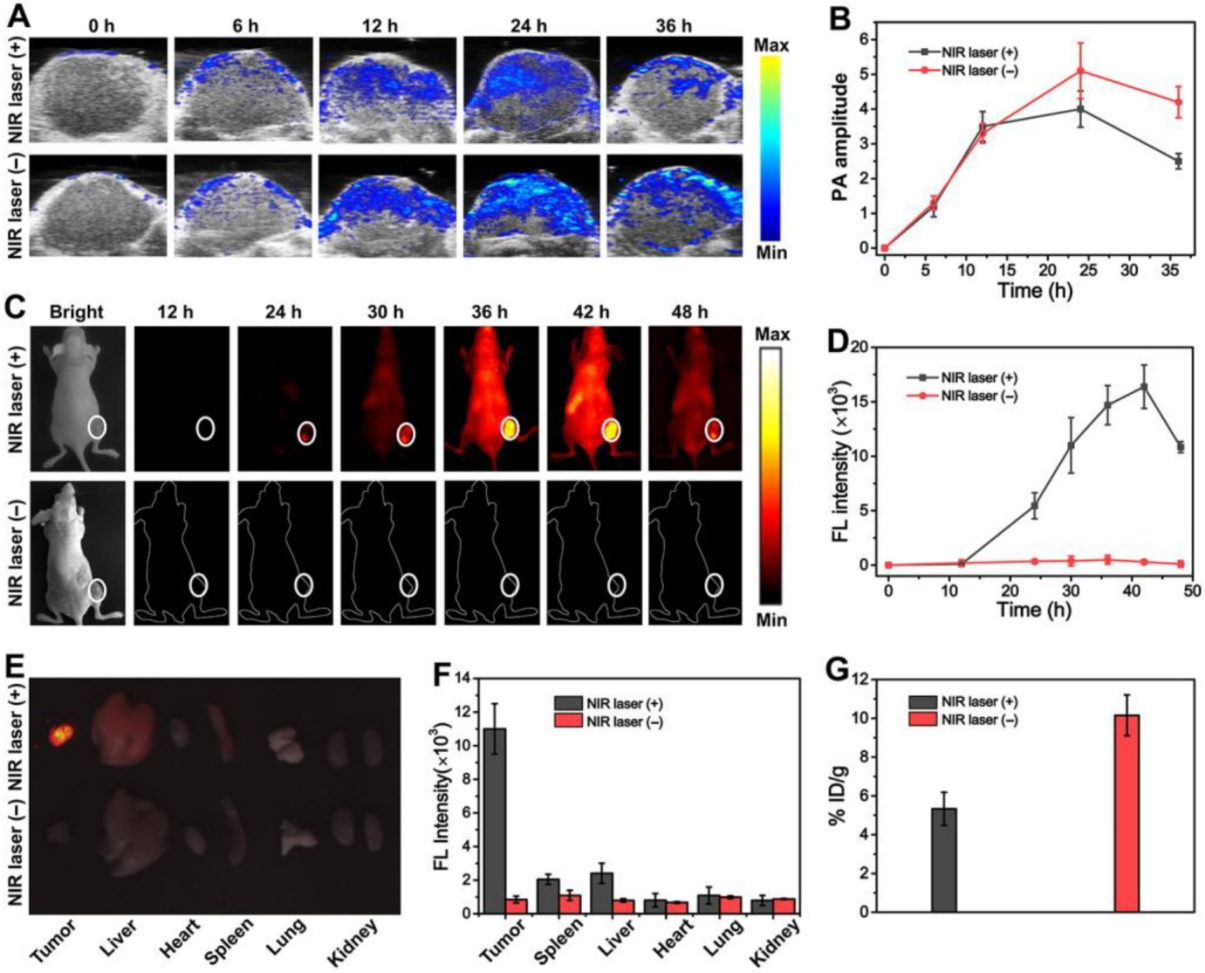
*In vivo* PA images **(A)** and PA amplitude **(B)** of tumors in MCF-7 tumor bearing mice treated with the AuNR@PEG/PolyRu Ves at different postinjection time points. *In vivo* NIR-II FL images **(C)** and time-dependent curves of fluorescence intensity **(D)** of the mice treated with AuNR@PEG/PolyRu Ves and with or without laser irradiation. Representative *ex vivo* NIR-II FL imaging **(E)** and quantitative fluorescence intensity** (F)** of main organs of MCF-7 cancer xenograft at 24 h post injection of AuNR@PEG/PolyRu Ves with and without NIR laser irradiation. (Power density of laser is 180 mW cm^-2^, exposure time is 900 ms) **(G)** The tumor retention efficiency of the AuNR@PEG/PolyRu Ves with or without NIR laser irradiation in tumor at 36 h post-injection.

Responsive dual NIR-II PA and FL imaging performance of experimental AuNR@PEG/PolyRu Ves were further investigated using an MFC-7 tumor animal model *in vivo*. To verify the stability of AuNR@PEG/PolyRu Ves in different physiological solutions including water, PBS, FBS, and cell culture medium, the AuNR@PEG/PolyRu Ves was dispersed in the abovementioned solution and no aggregation was observed after 5 days of incubation **(Figure S15)**. The AuNR@PEG/PolyRu Ves dispersed in PBS solution (200 μL, 4 mg mL^-1^) was intravenously injected into tumor xenograft nude mice *via* the tail vein. The mice injected with the vesicle were divide into two groups. As shown in **Figure 5A**, after injecting AuNR@PEG/PolyRu Ves without NIR laser irradiation, NIR-II PA signal was detected at the tumor site at 6 h postinjection. Apparently, NIR-II PA signal intensity continued to rise until 24 h and then PA signal intensity gradually decreased. The other group was treated with 808 nm laser irradiation (50 mW cm^-2^, 5 min) for 24 h. The trend of PA signal was consistent with the control group for 24 h, after which weaker PA signals were observed compared with the control group. The trend curve for the average PA signal intensity at 1240 nm at the tumor site with or without NIR laser irradiation was recorded **(Figure 5B).** A decrease in PA signal intensity was observed after NIR laser irradiation. This phenomenon was mainly explained by the disassembly of AuNR@PEG/ PolyRu Ves under NIR laser irradiation. The result implied that AuNR@PEG/PolyRu Ves was gradually accumulated in the tumor region *via* the enhanced permeability and retention (EPR) effect and could be slowly cleared with time in physiological environment 46. Thus, the synergistic influence of metabolization and accumulation led to the aforementioned result. However, when the tumor site was irradiated and AuNR@PEG/PolyRu Ves was triggered, AuNR@PEG/PolyRu Ves was disassembled into single AuNRs and Ru-complexes. Importantly, all results suggest that the appropriate time point for starting treatment with laser irradiation is 24 h after AuNR@PEG/PolyRu Ves injection. Further, the change in PA signal can mark the release of Ru-complexes *in vivo*.

To investigate AuNR@PEG/PolyRu Ves as NIR-II fluorescent nanoprobes *in vivo*, NIR-II fluorescence imaging was also detected at the same time. Therefore, time-dependent whole-body NIR-II fluorescence images were observed. As shown in **Figure 5C**, no fluorescence signal was detected in the tumor in the control group, indicating that the fluorescence of IR 1061 was quenched. The fluorescence signal significantly increased at the tumor site at 24 h post-injection after NIR laser pre-irradiation, as the fluorescent molecular IR1061 was released from AuNR@PEG/PolyRu Ves and its fluorescence signal was recovered. The fluorescence intensity was recorded and obviously increased after NIR laser irradiation at 24-h post-injection **(Figure 5D)**. Therefore, this result suggested that AuNR@PEG/PolyRu Ves emerged to be responsive to NIR light *in vivo*. In addition, the change of fluorescence signal after NIR laser irradiation could monitor the disassembly process of the AuNR Ves and the release of Ru-complex. To further study NIR-II imaging in tumors and major organs, mice were sacrificed at 24 h post-injection with or without NIR laser irradiation and tumors as well as other major organs was excised for NIR-II imaging **(Figure 5E)**. *Ex vivo* NIR-II imaging showed superior fluorescence brightness in the tumor compared with normal organs. Varying degrees of fluorescence signals were detected in the liver, spleen, and lung. Then, the fluorescence intensities of the tumor and organs were recorded **(Figure 5F)**. Nevertheless, a faint fluorescence signal was observed without NIR laser irradiation. The tumor-to-liver ratio in the AuNR@PEG/PolyRu Ves+NIR laser group was much higher than that in the AuNR@PEG/PolyRu Ves without NIR laser irradiation group. In addition, AuNR@PEG/PolyRu Ves also showed considerably high target-to-nontarget ratio, in agreement with the result of whole-body NIR-II FL imaging. The tumor retention efficiency of AuNR@PEG/PolyRu Ves with or without NIR laser irradiation was calculated by testing the Au content via inductively coupled plasma-mass spectrometry (ICP-MS) method **(Figure 5G)**. Consistently, the retention efficiency of the responsive AuNR@PEG/PolyRu Ves was 10.15% ID/ g in the tumor region at 36 h post-injection, which was two times higher than that of AuNR@PEG/ PolyRu Ves without NIR laser irradiation (5.33%) **(Figure 5G)**. This result is attributed to the size decrease of responsive AuNR@PEG/PolyRu Ves, resulting from the disassembly of AuNR@PEG/ PolyRu Ves under NIR laser irradiation and the decrease in the retention capability in the tumor region. Thereby, AuNR@PEG/PolyRu Ves could be used as NIR light-activatable optical nanoprobe to sensitively and specifically monitor the precise release of Ru-complex by NIR-II FL imaging. Briefly, NIR light response of NIR-II FL signal increase and NIR-II PA signal decrease reveals that AuNR@PEG/PolyRu Ves can precisely guide synergistic treatment because of its novel NIR light-activatable imaging properties. Dual-mode active NIR-II PA and FL imaging could by highly informative *in vivo*, which is essential for cancer imaging and determination of an accurate therapy.

### *In Vivo* Synergistic Chemo-PDT Cancer Therapy

**Figure 6:**
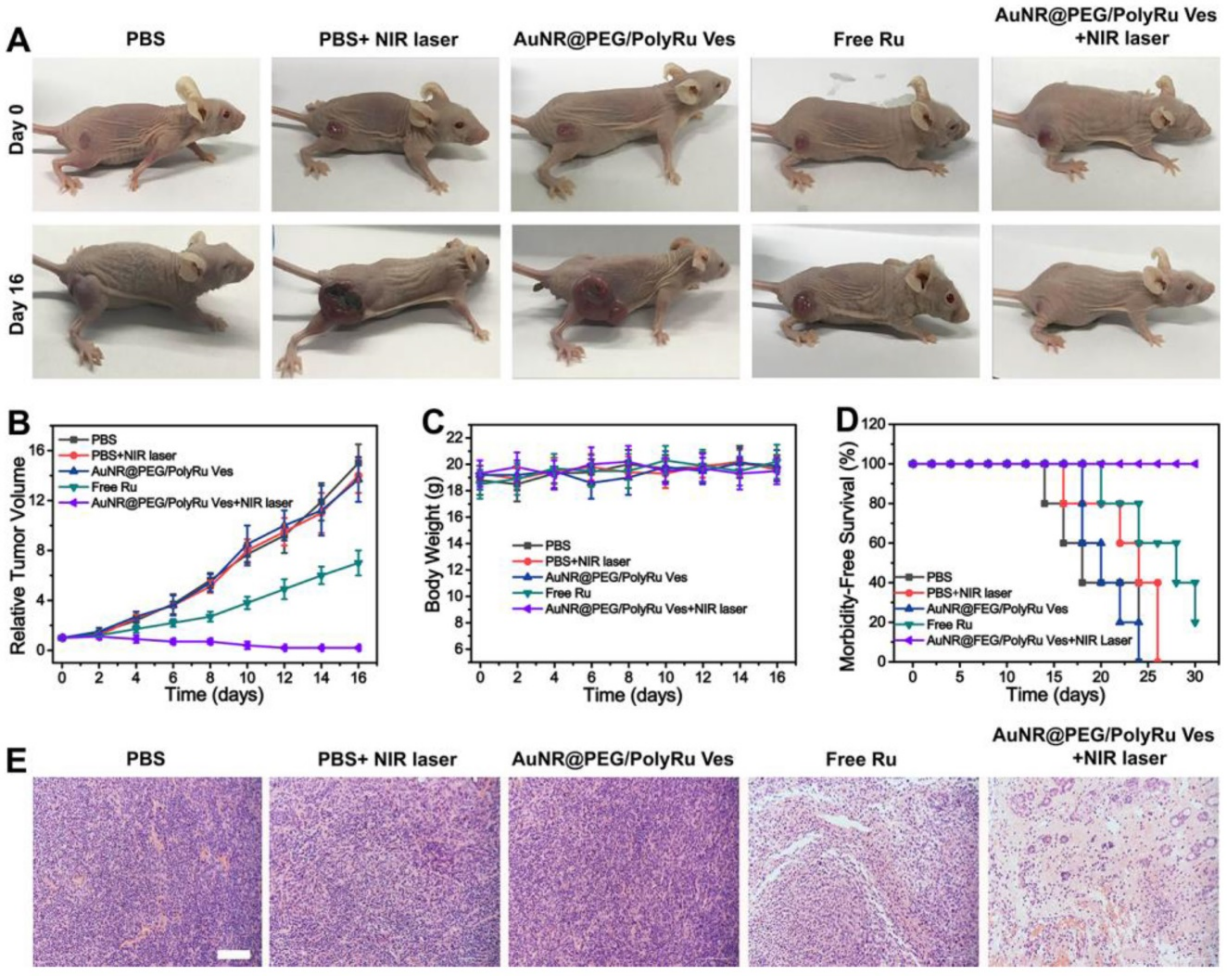
Syngernistic chemo-PDT of MCF-7 tumors *in vivo*. **(A)** Photographs of tumor-bearing mice in different therapy groups. **(B)** Tumor growth curves of mice after intravenous injection of different formulations. **(C)** The body weight of tumor-bearing mice in different treatment groups. **(D)** Animal survival curves after different treatments. **(E)** Images of hematoxylin and eosin (H&E) staining of tumor sections in different treatment groups. Scale bars: 100 µm.

Encouraged by the excellent *in vitro* therapy effect and *in vivo* responsive NIR-II PA and FL imaging results, we further studied anticancer effect of the AuNR@PEG/PolyRu Ves *in vivo*. We first established nude mice bearing a tumor after MCF-7 inoculated into the right leg of the mice. Before cancer treatment, AuNR@PEG/PolyRu Ves (100-1600 μg mL^-1^) were incubated with blood from BALB/c nude mice for hemolysis analysis to detect their* in vivo* biocompatibility. The results of this analysis showed that AuNR@PEG/PolyRu Ves has excellent biocompatibility **(Figure S16)**. The good blood compatibility and nontoxic features of AuNR@PEG/ PolyRu Ves demonstrated that the design of PolyRu overcame the side effects of conventional photocaged Ru-complex for *in vivo* applications. For cancer therapy, the tumor-bearing mice were randomly divided into five groups (five mice in each group), including PBS, PBS+NIR laser, free Ru, AuNR@PEG/ PolyRu Ves, and AuNR@PEG/PolyRu Ves+NIR laser. The samples were intravenously injected *via* the tail vein into tumor-bearing mice on the first and third days of the experiment. Subsequently, the tumor site was irradiated with 808 nm laser (50 mW cm^-2^, 5 min) at 24 h after injection (AuNR@PEG/PolyRu Ves+NIR laser group). In the other three control groups, the relative tumor volumes were 7.5 to 14 times larger after 16 days **(Figure 6A, B)** compared with the initial volumes. The tumors in control groups grew quickly in the following days. In the free Ru-complex group, the tumor size was inhibited because of limited treatment effect of free Ru-complex as chemotherapeutic drugs. Compared with the three control groups, the AuNR@PEG/PolyRu Ves+NIR laser group showed more significant inhibition of tumor growth. This result indicated that the synergistic photoactivated PDT and chemotherapy was effectively suppressing tumor growth. The body weights of the mice were also recorded for every 2 days during the treatment process** (Figure 6C)**. No decrease in the body weights of the mice was observed during the treatment process, indicating that the injection of AuNR@PEG/PolyRu Ves nanoparticles and NIR laser irradiation have minimal side effects on the mice. More importantly, most the AuNR@PEG/PolyRu Ves were cleared out from mice at day 12 **(Figure S17)**. Moreover, animals in the free Ru-complex and three control groups had an average life span of 18-25 days, whereas animals in the AuNR@PEG/PolyRu Ves +NIR laser group survived over 30 days without a single mice death after synergistic treatment **(Figure 6D)**. As displayed in **Figure 6E**, H&E staining images of the tumor tissues after treatment for 16 days showed that a number of cancer cells in the AuNR@PEG/PolyRu Ves+NIR laser group were killed and most cancer cells in the saline group survived. A limited therapy effect of free Ru treatment was observed. These results demonstrated a good antitumor performance of AuNR@PEG/PolyRu Ves under NIR laser irradiation.

The main organs (heart, liver, lung, spleen, and kidney) of mice in the five groups were evaluated by H&E staining **(Figure S18)**. Compared with the organs in the control group, the organs in the AuNR@PEG/PolyRu Ves+NIR laser group did not show any pathological tissue damage or abnormality. This phenomenon showed the remarkable biocompatibility of AuNR@PEG/PolyRu Ves and minimal side effects. All of results confirmed that synergistic chemo-photodynamic treatment using AuNR@PEG/PolyRu Ves eliminated substantial systemic toxicity *in vivo*.

## Conclusion

In summary, we have developed a NIR light-activatable AuNR vesicle loaded with NIR-II FL dye for NIR-II PA and FL dual-mode imaging guiding the release of photosensitizer and chemotherapeutic Ru-complexes for cancer theranostics. The amphiphilic AuNR was coated with PEG and light-responsive polyprodrug of PolyRu. The vesicles can be triggered by NIR light with a side chain-cleavage scatter, leading to the disassembly of Ves, generation of ^1^O_2_ and release of Ru complex. The obvious change of NIR-II PA and FL signals makes it possible to monitor the NIR light activation of the AuNR Ves. Additionally, the combination of chemotherapy and PDT verified an efficient antitumor effect of the Ves *in vitro* and *in vivo*. The AuNR Ve-based system possessing not only precisely controlled drug release but also the intrinsic ability to monitor prodrug activation and active drug release offers a new opportunity for the development of theranostic nanomedicine to maximize therapeutic benefits.

## Methods

### NIR Light Responsiveness of AuNR@PEG/PolyRu Ves and Drug Release

Aqueous solution of AuNR@PEG/PolyRu Ves were pre-irradiated under 808 nm laser (50 mW cm^-2^) for 5 min. The morphology change of AuNR@PEG/ PolyRu Ves after irradiation for 15 min and 30 min was observed by transmission electron microscopy (TEM). The vesicle size change with time was monitored *via* dynamic light scattering (DLS). The change in the fluorescence spectra and fluorescence images in 96-well plate were observed to study the NIR light responsiveness of AuNR@PEG/PolyRu Ves. AuNR@PEG/PolyRu Ves (3.0 mg) was dissolved in PBS (pH 7.4, 1.0 mL). The solution was placed into a pre-swelled dialysis tube (MWCO= 3000), and immersed in the buffer solution (19 mL). Afterwards, the solution was incubated in a bath shaking incubator at 37°C. The solution was without NIR laser irradiation or 5-min pre-irradiation (808 nm, 50 mW cm^-2^). Subsequently, 3 mL of buffer solution outside of the dialysis tube was removed at a preset time to record the UV-vis spectra at 560 nm. After sampling, 3 mL of fresh buffer solution was supplemented to the dialysate. The release of Ru-complexes was calculated according to the standard curve.

### Detection of ^1^O_2_

The FL signal change of DPBF solution (V_methanol_/V_H2O_: 1/1, 150 mM ) was used to detect ^1^O_2_. AuNR@PEG/PolyRu Ves (50 μg mL^-1^) was added into the solution of DPBF and then treated with NIR laser irradiation (50 mW cm^-2^). Finally, the fluorescence signal at 350 nm of solution was tested.

### *In vitro* cytotoxicity of the AuNR@PEG/PolyRu Ves

Human breast adenocarcinoma MCF-7 cell line and 4T1 cells proved by American Type Culture Collection (ATCC) were cultured in RPMI 1640 medium (ATCC, Manassas, VA) containing 10% fetal bovine serum (10%, v/v), and 1% penicillin/ streptomycin. All the cells were cultured at 37°C, 95% humidity and 5% CO_2_ in an incubator.

A standard CCK-8 assay was performed for *in vitro* evaluation*,* in accordance with the manufacturer's protocol. To explore the cytotoxicity of Ves with or without irradiation, the cells were seeded into a 96-well cell culture dish (5×10^3^ cells each well) and incubated for 24 h. Subsequently, different concentrations of Ru-complex and AuNR@PEG/ PolyRu Ves were added into the well, and incubated with cells for 12 h. For the irradiation groups, cells were irradiated with NIR laser (808 nm, 50 mW cm^-2^) for 5 min. Following this, all cells were washed with PBS and cultured in fresh medium for another 12 h. After that, cells were stained with 10 μL CCK-8 for 5 min. The absorbance was then observed at 450 nm. The viability of cells was determined using the following formula: Viability (%) = (mean of absorbance value of treatment group/mean absorbance value of control) ×100%.

### Cell Apoptosis

MCF-7 cells were seeded in 6-well plates (1 × 10^6^ cells each well) for 12 h. Then, cells were cultured with the sample (200 μg mL^-1^) for 12 h and then treated with NIR laser irradiation (808 nm laser, 50 mW cm^-2^) for 5 min. All experiments were randomly divided into four groups (free Ru, PBS, AuNR@PEG/ PolyRu Ves without NIR laser irradiation, AuNR@PEG/PolyRu Ves with NIR laser irradiation). After incubation at 37 °C for 6 h, cells were collected and suspended in 195 µL of binding buffer. Then, 5 µL of annexin V-FITC and 10 µL of PI working solution were added. And the suspension of cells were incubated for 30 min at 37 °C, and then immediately tested by flow cytometry.

### Establishment of Animal Tumor Model

All nude mice were maintained under required conditions and had free access to food and water throughout the experiments. The MCF-7 BALB/c nude mice models were developed by a subcutaneous injection of MCF-7 cells (5 × 10^6^ cells) into the right legs of the mice. The Animal Ethics Committee of Fujian Medical University of China approved the study protocol. BALB/c nude mice (5-6 weeks old, ∼20 g) were purchased from Shanghai SLAC laboratory Animal Co., Ltd. (Shanghai, China).

### *In vivo* NIR-II PA imaging and FL imaging of the AuNR@PEG/PolyRu Ves

Mice bearing subcutaneous MCF-7 tumors with a tumor volume of 60∼100 mm^3^ were randomly divided into two groups. Next, AuNR@PEG/PolyRu Ves (200 μL, 4 mg mL^-1^) was injected intravenously. One group was exposed to NIR laser irradiation (808 nm, 50 mW cm^-2^) for 5 min at the tumor site after 24 h, whereas the other group was maintained without NIR laser irradiation throughout. Time-dependent whole- body NIR-II fluorescence images were obtained by *in vivo* imaging (808 nm laser, exposure time: 900 ms). The excitation was obtained by an 808 nm laser. The 808 nm laser was filtered with 808 nm long-pass filters. Fluorescence emission was collected at 1150 nm with a 1150 nm band-pass filter. Then, the tumors were excised for *ex vivo* fluorescence imaging at 24 h. For *in vivo* photoacoustic imaging, MCF-7 tumor- bearing mice were used. AuNR@PEG/PolyRu Ves (200 μL, 4 mg mL^-1^) in PBS solution was intravenously injected via the tail vein into the MCF-7 tumor-bearing nude mice. Simultaneously, the whole tumor region of mice was scanned using a Visual Sonic Vevo 2100 LAZR system equipped with a 40 MHz, 256-element linear array transducer as a function of time. PA imaging was conducted using a 1240-nm laser and the images were constructed using the Visual Sonic Vevo software. During all tests, mice were anesthetized with isoflurane along with oxygen using an anesthesia system.

### *In vivo* Antitumor Efficacy

MCF-7 BALB/c nude mice with a tumor volume of approximately 100 mm^3^ were randomly divided into five groups, including PBS, PBS+NIR laser, Free Ru, AuNR@PEG/PolyRu Ves, and AuNR@PEG/ PolyRu Ves+NIR laser, and the initial weight and tumor volume were recorded. Next, AuNR@PEG/ PolyRu Ves was injected intravenously and irradiated with 808 nm laser (50 mW cm^-2^, 5 min). Because maximum tumor accumulation of the nanoparticle was observed at 24 h post-injection, the laser irradiation time point of 24 h was selected. The other group was injected with AuNR@PEG/PolyRu Ves was maintained without NIR laser irradiation. Tumor volume was measured every two days and was calculated using the following equation: tumor volume = length × width × width/2. The body weight and survival status of mice was recorded.

### Histological Analysis

The major tissues and tumors at excised at the end of the antitumor efficacy experiment, followed by fixing with 4% formaldehyde and embedding in paraffin. Then, the tissues were cut into slices of 2 μm thickness for H&E and Tunnel. Then, the stained samples were observed by microscope.

## Supplementary Material

Supplementary materials and methods, figures.
