# Supplementary material for: Light-activated gold nanorod vesicles with NIR-II fluorescence and photoacoustic imaging performances for cancer theranostics
Source: Theranostics. 2020 Mar 26;10(11):4809–21. doi: 10.7150/thno.44376 (PMC7163452; doi:10.7150/thno.44376)
Supplement: Supplementary file 1 — Supplementary materials and methods, figures. [file thnov10p4809s1.pdf]

Supporting information:

Light-activated gold nanorod vesicles with NIR-II fluorescence and photoacoustic imaging performances for cancer theranostics

*Xiaoguang Ge, Qinrui Fu, Lichao Su, Zhi Li, Wenmin Zhang, Tao Chen, Huanghao Yang, Jibin Song**

Materials. Ruthenium (III) chloride trihydrate, 2,2′:6′,2″-terpyridine (98%), 2,2′ biquinoline (98%), silver hexafluorophosphate (98%), potassium hexafluorophosphate (99.5%), N, N, N′, N″, N″-pentamethyldiethylenetriamine (99%, PMDETA), CuBr (98%), tert-butyl methacrylate (98%, contains 200 ppm monomethyl ether hydroquinone as inhibitor), 2-hydroxyethyl disulfide, α-bromoisobutyryl bromide (98%), anisole, 4-(2-{2-chloro-3-[2-(2,6-diphenyl-4H-thiopyran-4-ylidene)ethylidene]cyclohex-1-en-1-yl}ethenyl)-2,6-diphenyl-1λ^4-thiopyran-1-ylium; tetrafluoroboranuide (IR 1061, 80%), hydrogen tetrachloroaurate (III) trihydrate ($HAuCl_4{\cdot}3H_2O$), cetyltrimethylammonium bromide (CTAB, 98%), and sodium borohydride (96%) silver nitrate (99%) were purchased from Sigma Aldrich. 4-hydroxybenzonitrile (98%), triethylamine (99%), thionyl chloride (99%),

trifluoroacetic acid (99.5%), 6-chlorohexanol (95%), potassium iodide (99%) were purchased from Aladdin. Poly (ethylene glycol) methyl ether thiol (SH-PEG, average Mw 5000) was purchased from Ruixi Biological Technology (Xi'an, China). Ultrapure water (18.25 MΩ resistivity, 25 °C) was used in all experiments. 2,2,6,6-tetramethylpiperidine (TEMP) was purchased from J&K Scientific Ltd (Beijing, China). Cell Counting Kit-8 (CCK-8) was purchaseed from MedChemExpress (Monmouth Junction, NJ, USA). Propidium iodide (PI) dye and Annexin V-FITC apoptosis detection kit were purchased from Beyotime Biotechnology (Shanghai China).

Equipments. Nuclear magnetic resonance (NMR) spectra were measured on a Bruker AVANCE III 500 in deuterated chloroform ($CDCl_3$) and deuterated dimethyl sulfoxide (DMSO-d6). TEM images were performed on an HT7700 transmission electron microscope (HITACHI, Japan) at 100 kV. DLS was collected through a Malvern Zetasizer Nano ZS (Malvern, U.K.). UH4150 spectrophotometer (HITACHI, Japan) have been used to record ultraviolet−visible−near-infrared light (UV−VIS−NIR) absorption spectra Fluorescence spectra were measured on Edinburgh FLS980 Spectrometer. The fluorescence images of cells were obtained on a confocal fluorescence microscope (Nikon C2). The fluorescence images *in vivo* were performed on *IN-VIVO* MASTER which provided by GRAND-IMAGING.

Photoacoustic imaging (PAI) was performed by the InVision 128 MSOT system (iThera Medical, Germany).

Synthesis of Ru(tpy)Cl$_3$. 0.5 mmol $RuCl_3 \cdot 3H_2O$ and 0.5 mmol 2,2′;6′,2″-terpyridine were dissolved in 70.0 mL ethanol. Then, the mixture was heated to 80 °C for 3 h under vigorous stirring. After that, the solution was cooled down to room temperature. The brown powders were collected by filtration. The product was washed with ethanol and diethyl ether for three times. Finally, the product was dried for next reaction. (yield :75%)

Synthesis of [Ru(tpy)(biq)(Cl)]Cl. [Ru(tpy)(biq)(Cl)]Cl was synthesized according to the literature.[S1] 0.2 mmol 2,2’-biquinoline and 0.2 mmol Ru(tpy)Cl$_3$ were dissolved in 10.0 mL mixed solvent of H_2O amd ethanol with the volume ratio of 3:1. Ttrimethylamine was added after the mixture was bubbled with argon. After that, the mixture was refluxed for 10 h in the dark. Then, purple solution was obtained after filtration. Finally, the filtrate was evaporated under reduced pressure. The product was purified by column chromatography with silica gel (eluent: dichloromethane / methanol =8:1). (yield :30%) The solvent was evaporated and the product was obtained as violet powders.

Synthesis of [Ru(tpy)(biq)(H_2O)](PF_6)$_2$. [Ru(tpy)(biq)(H_2O)](PF_6)$_2$ was synthesized according to the literature.[S2] Brifely, 0.047 mmol [Ru(tpy)(biq)(Cl)]Cl and 0.1 mmol $AgPF_6$ were dissolved in 3:1 acetone/H_2O mixture (8.0 mL). The solution was degassed and heated under reflux in an argon atmosphere for 2 h. The solution was cooled and filtered to remove AgCl. The solvent of the reaction was reduced to ~2 mL. Then, an aqueous solution of KPF_6 was added. The precipitate was filtered, washed with H_2O, and dried to give a purple solid (62 mg, 73%).

Synthesis of 4-((6-hydroxyhexyl)oxy)benzonitrile(CPH). 60.0 mmol hydroxybenzonitrile, 60.0 mmol NaOH, 0.05 g KI were added into 70.0 mL ethanol. Then, 20.0 mL ethanol containing 0.07 mmol 6-chlorohexanol was added drop by drop before the mixture was refluxed for 6 h under argon atmosphere. The crude product was purified by column chromatography (eluent: petroleum ether/ ethyl acetate = 1.5/1). Finally, white waxy solid was obtained. (yield :95%). ^{1}H NMR (400 MHz, $CDCl_3$, TMS) δ (ppm): 7.60 (d, 2.01H, J = 7.5 Hz), 6.95 (d, 2H, J = 7.5 Hz), 3.99 (t, 2.02H, J = 7.5 Hz), 3.68 (t, 2H, J = 7.5 Hz), 1.80 (m, 2H), 1.62(m, 2H), 1.46 (m, 4.01H).

Synthesis of Initiator of Bis[2-(2′-bromoisobutyryloxy)ethyl]disulfide (DTBE). To synthesize PAA-S-S-PAA, initiator DTBE containing disulfide bond was first prepared. 5.0 mmol hydroxyethyl disulfide and 30.0 mmol triethylamine were dissolved in 30.0 mL dichloromethane with stirring in ice bath. Afterwards, 14.0 mmol α-bromoisobutyryl bromide in dichloromethane was added dropwise. The reaction lasted at 0 °C for 1 h, following the mixture continue for 24 h at room temperature. The reaction mixture was washed with saturated salt water and distilled water three times. The organic phase was dried over anhydrous sodium sulphate, filtered and the dichloromethane evaporated. The brown viscous liquid was obtained. (yield: 95%).

Synthesis of Polymer Containing Cyano Group of the PCPH. Firstly, PAA-S-S-PAA was prepared through atom transfer radical polymerization (ATRP) approach. In a typical experiment, 23.0 mg DTBE, 28.0 μl PEMDETA and 2.2 mL tertiary-butyl methacrylate were dissolved in 2.0 mL anisole. The mixture was bubbled with argon for 20 min. In the following, 17.0 mg CuBr was added quickly and again bubbled with argon for 10 min. The mixture reacted at 100 °C with stirring for 12 h. Then, the reaction was quenched by adding 100 ul methanol and diluted in 10.0 mL dichloromethane. To remove redundant copper species, the mixed solution was purified through a column chromatography. Then, 0.4 mL trifluoroacetic

acid (TFA) was added into above resulting solution and reacted for 12 h at room temperature to removal the tert-butyl protecting groups. The resulting mixture was concentrated by evaporation and added into n-hexane. White solid appeared immediately and was collected by filtration. The purified product (PAA-S-S-PAA) was obtained. (yield :70%)

To synthesis of PCPH grafted with cyano group, the CPH was covalently attached with PAA. Typically, 1.0 g PAA was dissolved in 10.0 mL thionyl chloride and refluxed for 2 h. After that, the solution was treated by vacuum distillation to remove the solvent. The above reacted product was dissolved in 10.0 mL CH_2Cl_2. The solution was added dropwise into dichloromethane containing 3.0 g CPH and 10.0 mL TEA in the ice bath. The mixed solution kept the reaction at 0 °C for 1 h and another 12 h at room temperature. The polymer of PCPH was precipitated in ethyl alcohol and the precipitates were dried in vacuum oven.

Preparation of the Gold Nanorod (AuNR). 0.37 g cetyltrimethylammonium bromide (CTAB) was added in 10.0 mL deionized water under stirring at 30 °C until the mixture became clear, followed 200 μL gold (III) chloride trihydrate ($HAuCl_4·3H_20$) (20 mg mL^{-1}) was introduced. Then, ice water containing 0.4 mg mL^{-1} $NaBH_4$ and 0.4 mg mL^{-1} NaOH was added. Another 15 min was repaired to complete the reaction. Another round-bottomed flask was prepared, followed 1.48 g CTAB was dissolved in 40.0 mL deionized water. Then, 200

μL gold (III) chloride trihydrate (20 mg mL^{-1}) was added. After that, 0.28 mL $AgNO_3$ (0.1 M aqueous) and 2.0 mL hydroquinone (0.1 M aqueous) were added successively. Afterwards, 4.8 mL solution initially prepared was added. The mixture reacted for 1.5 h at 30 ℃. Ultimately, the AuNR modified by CTAB (AuNR@CTAB) was purified by centrifugation and washed with deionized water for three times.

Synthesis of AuNR Coated with PEG and Photoactivated Polymer (AuNR@PEG/PolyRu).

To prepare amphiphilic gold nanorods attached with PEG and PolyRu. AuNR@CTAB (50 nM) was mixed with 0.1 mL of 2-(2-aminoethoxy)ethanol and the mixture was stirred for 24 h. The modified AuNRs were purified by centrifugation at 9000 g for 10 min and further dispersed in 5 mL of DMSO. Amphiphilic AuNR@PEG/ PCPH was synthesized by a "grafting to" reaction. Briefly, the mixture of thiolated PEG (PEG-SH, Mn = 5000) and thiolated PCPH was slowly added into the modified AuNR dispersion, and the solution was stirred for 12 h. The AuNR@PEG/P PCPH was purified by centrifugation (10,000 g, 15 min) and dispersed in 5 mL of acetone. To synthesize photoactivated AuNR@PEG/PolyRu containing Ru complexes, AuNR@PEG/P PCPH in acetone. Excessive

$[Ru(tpy)(biq)(H_2O)](PF_6)_2$ was added and stirred for 12 h in the dark. Finally, AuNR@PEG/PolyRu were purified by centrifugation and odispersed in chloroform.

Preparation of the Assembly of Photoactivated Gold Nanorod Vesicle (AuNR@PEG/PolyRu Ves). To prepare NIR-II dye loaded AuNR@PEG/PolyRu Ves, AuNR@PEG@PolyRu (2.0 mg) and IR 1061 (20.0 μg) were first dissolved in chloroform. In order to prepare the aqueous phase for microemulsion, 50.0 mg of PVA (MW: 9000–10000), as a polymer stabilizer, was dissolved in 5.0 mL of deionized (DI) water at 60 °C. After the PVA was completely dissolved, the clear solution was cooled to room temperature. The organic phase was then added to the PVA solution and emulsified for several minutes by pulsed sonication. The emulsion was then stirred at room temperature for 12 h to evaporate the chloroform. The resulting AuNR@PEG/PolyRu Ves were washed with DI water three times to remove excess PVA.

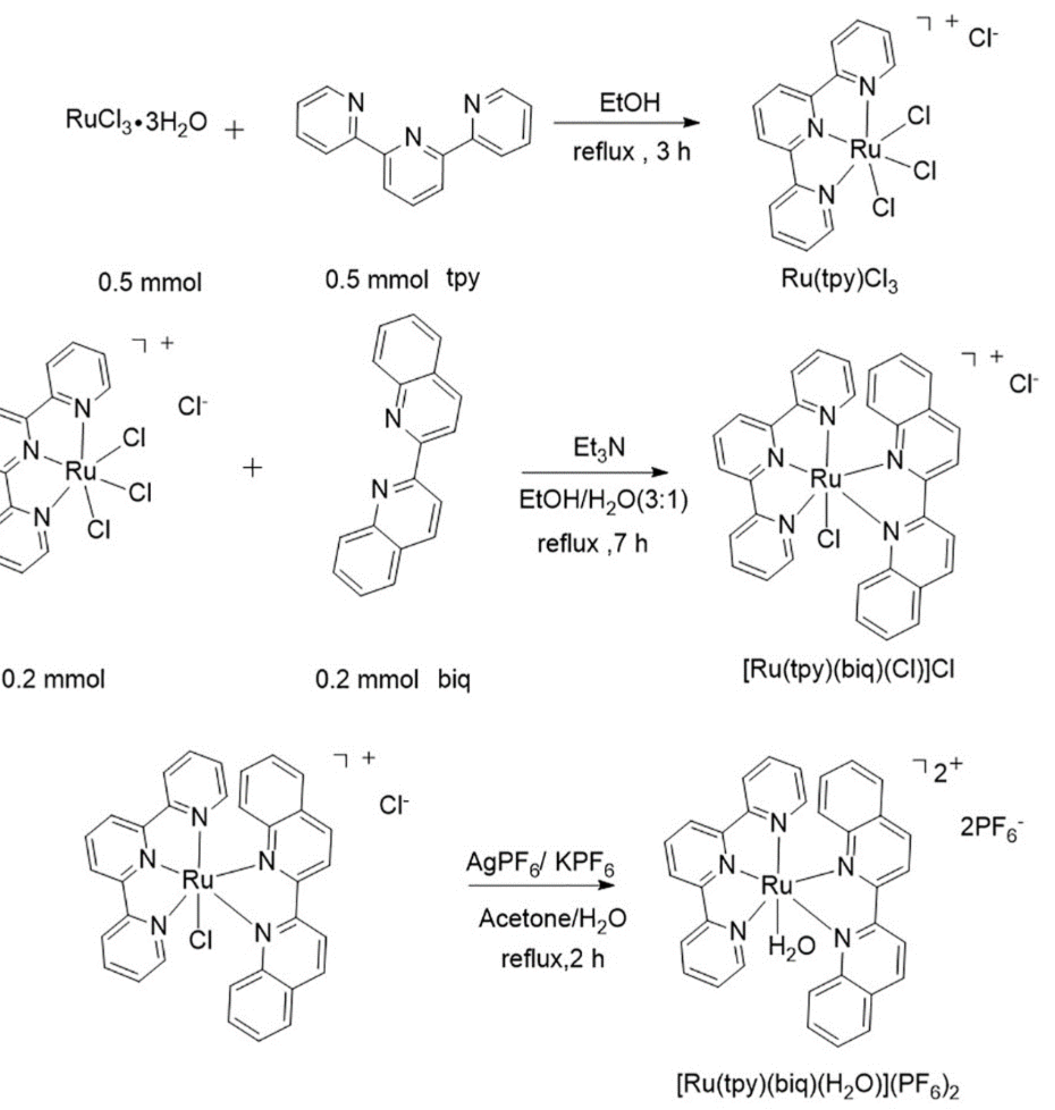

Figure S1. Synthesis of $[Ru(tpy)(biq)(H_2O)](PF_6)_2$.

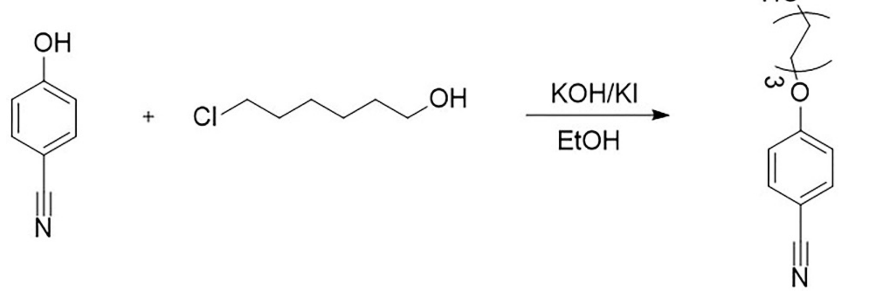

Figure S2. Synthesis of 4-((6-hydroxyhexyl)oxy)benzonitrile(CPH).

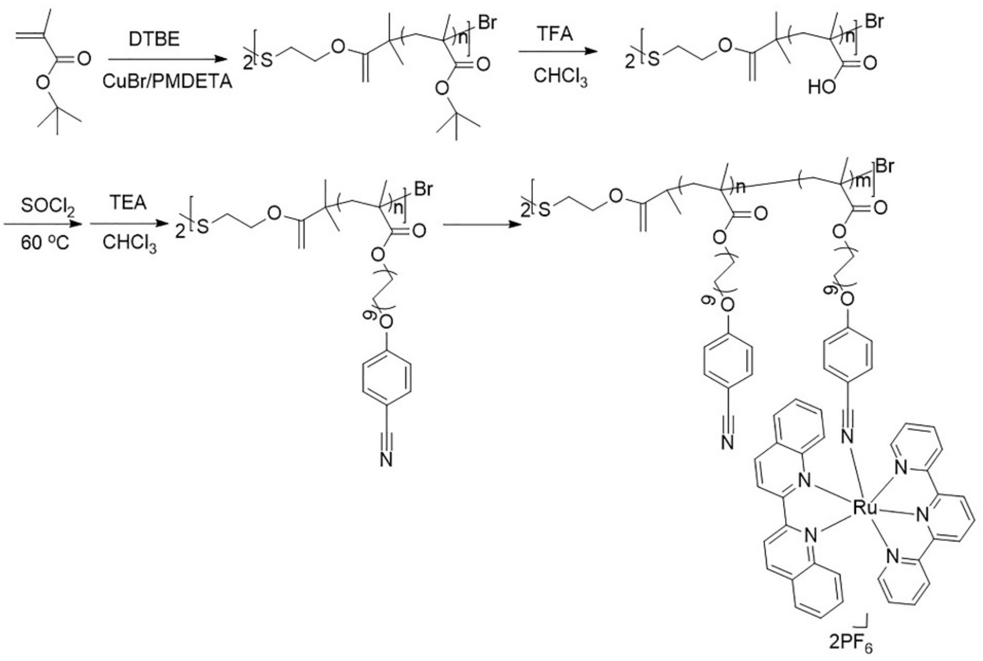

Figure S3. Synthesis of PolyRu.

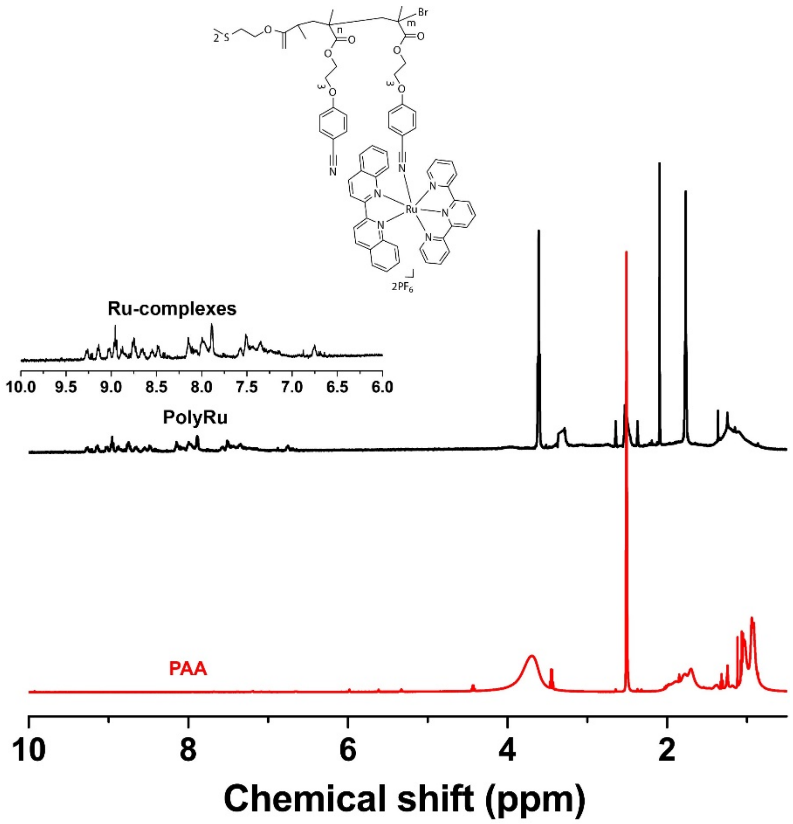

Figure S4. ^{1}H NMR spectra of PAA and PolyRu (400 MHz, DMSO-d6).

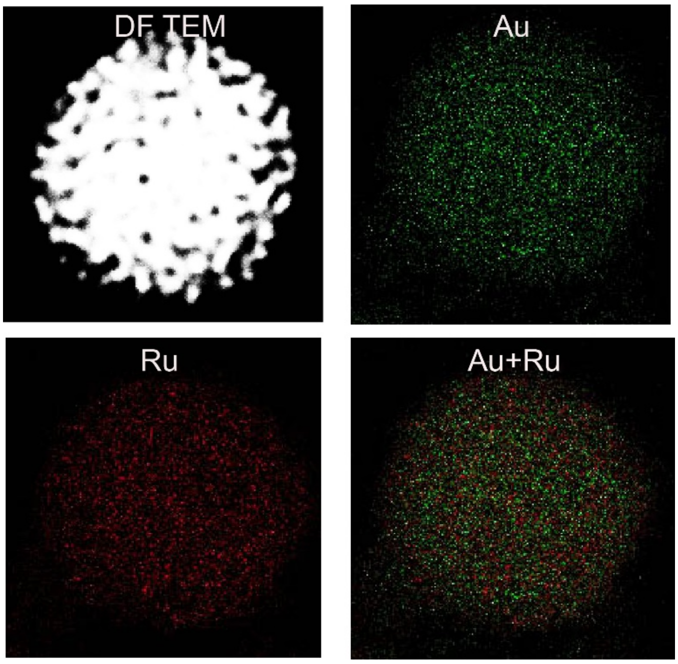

Figure S5. Dark-field TEM and element mapping (Au and Ru elements) of a AuNR@PEG/PolyRu Ve.

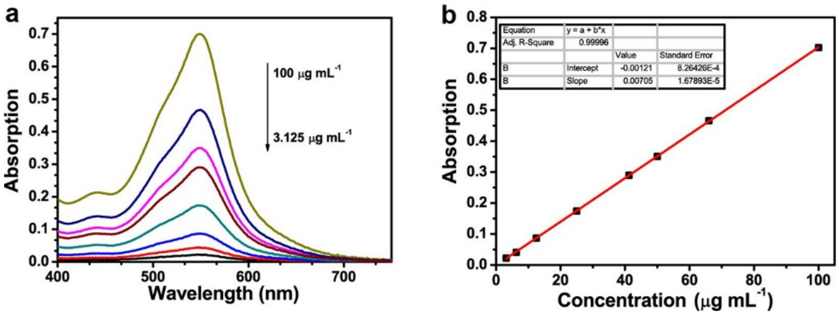

Figure S6. (a) UV-vis spectrum of different concentration of $[Ru(tpy)(biq)(H_2O)](PF_6)_2$. (b) the standard curve of $[Ru(tpy)(biq)(H_2O)](PF_6)_2$.

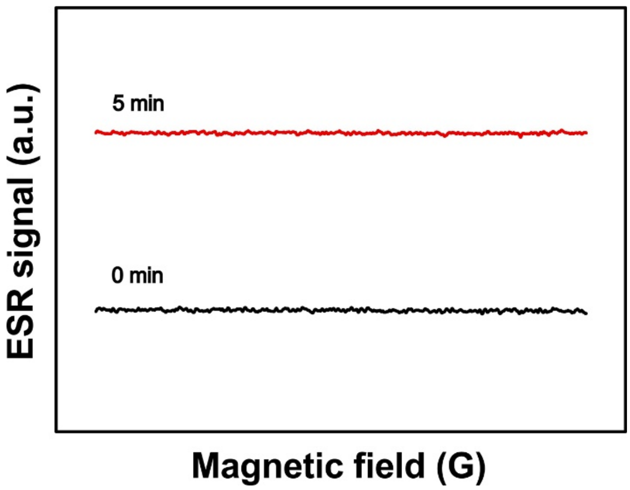

Figure S7. ESR spectra for $^1O_2$ of AuNR Ves under 808 nm laser irrradiation.

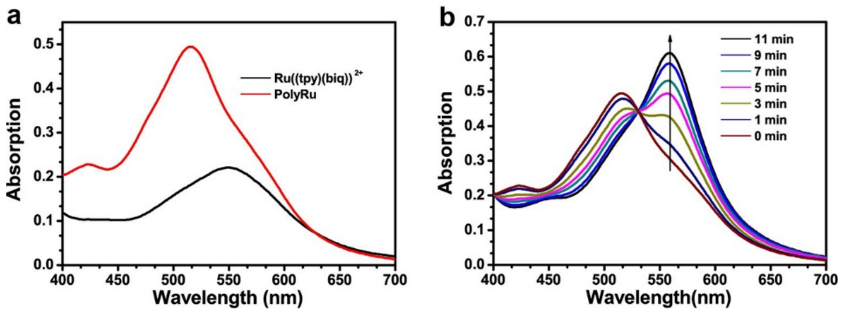

Figure S8. (a) UV-vis spectrum of PolyRu and $[Ru(tpy)(biq)(H_2O)](PF_6)_2$, (b) UV-vis spectrum of PolyRu with 660 nm laser irradiation.

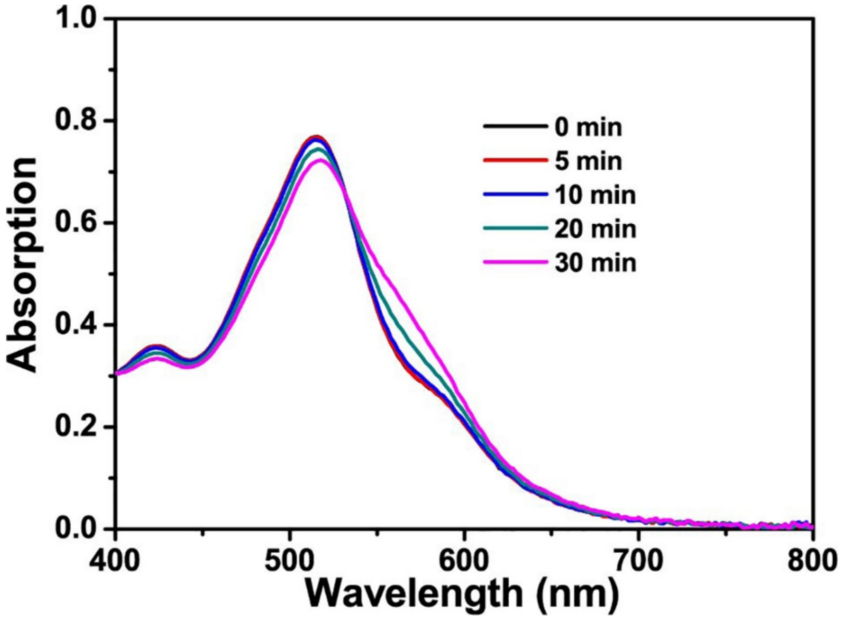

Figure S9. UV-vis spectrum of PolyRu with 808 nm laser irradiation.

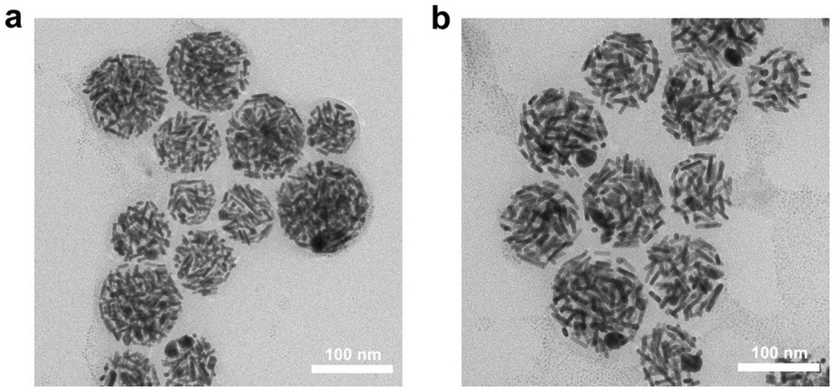

Figure S10. TEM images of AuNR@PEG/PolyRu Ves after NIR laser irradiation for (a) 2 min and (b) 5 min.

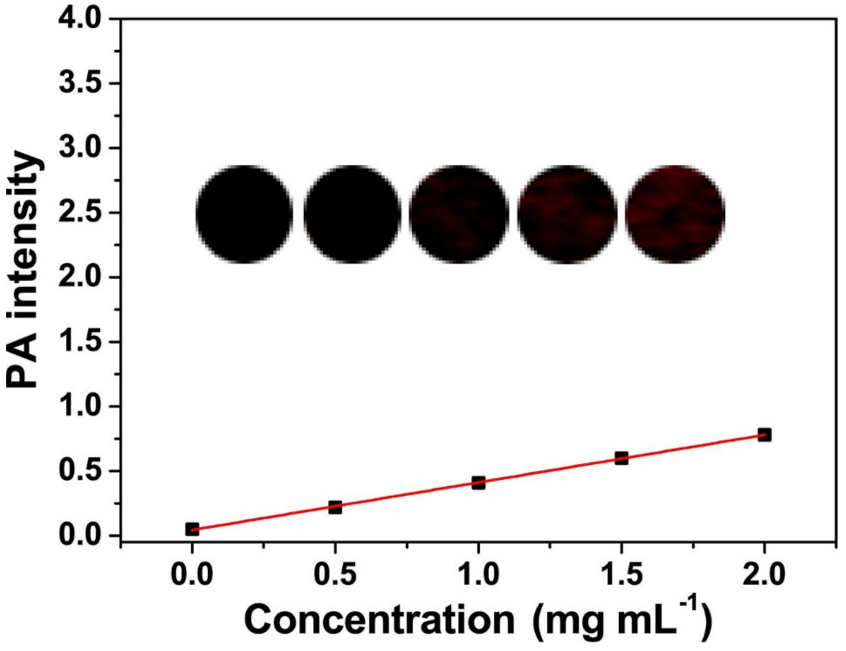

Figure S11. PA images of different concentration AuNR in water exposed with PA laser.

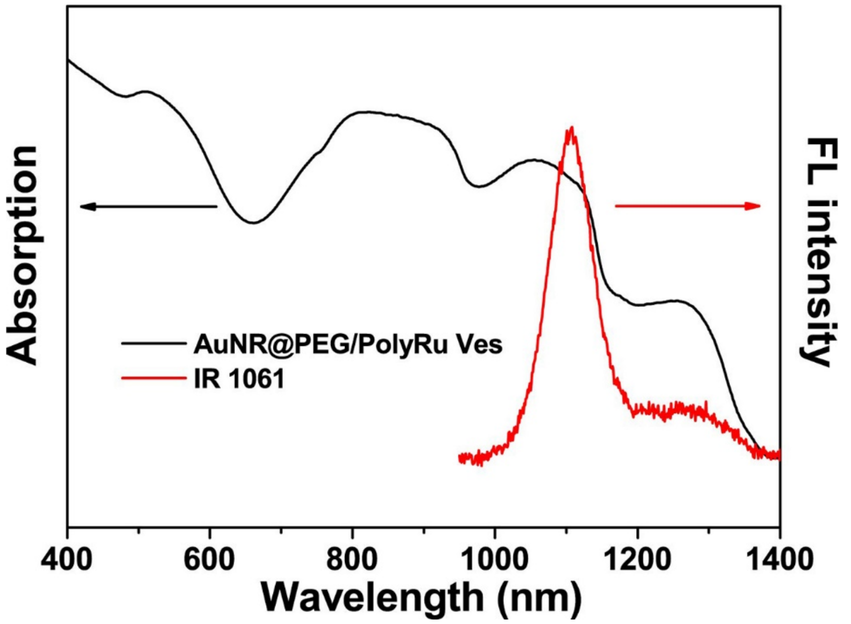

Figure S12. UV-vis spectrum of AuNR@PEG/PolyRu Ves (blcak line) and fluorescence spectrum of IR 1061 (red line).

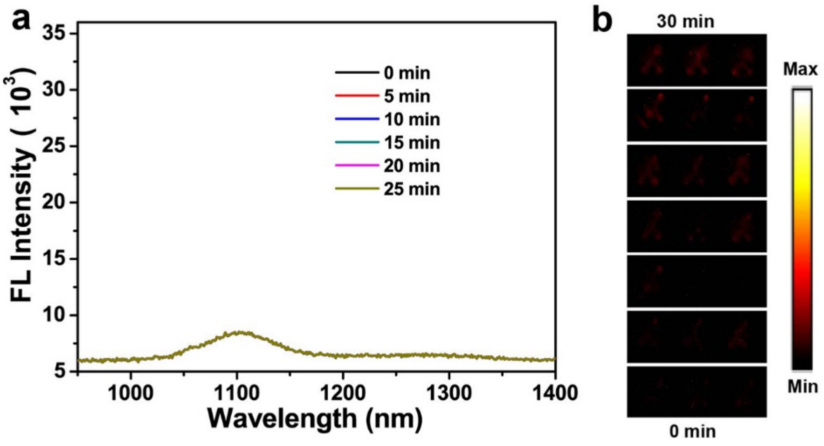

Figure S13. (a) Fluorescence spectra of AuNR@PEG/PolyRu Ves with NIR laser irradiation. (b) Fluorescence images *in vitro* with different treatment.

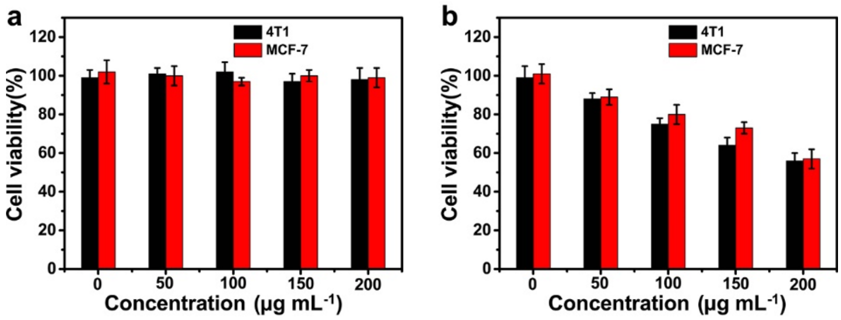

Figure S14. (a) Cell viability of 4T1 and MCF-7 treated with different concentration of AuNR@PEG/PolyRu Ves without NIR laser irradiation. (b) Cell viability of 4T1 and MCF-7 treated with different concentration of Ru-complexes with NIR laser irradiation (50 mW cm^{-2}, 5 min).

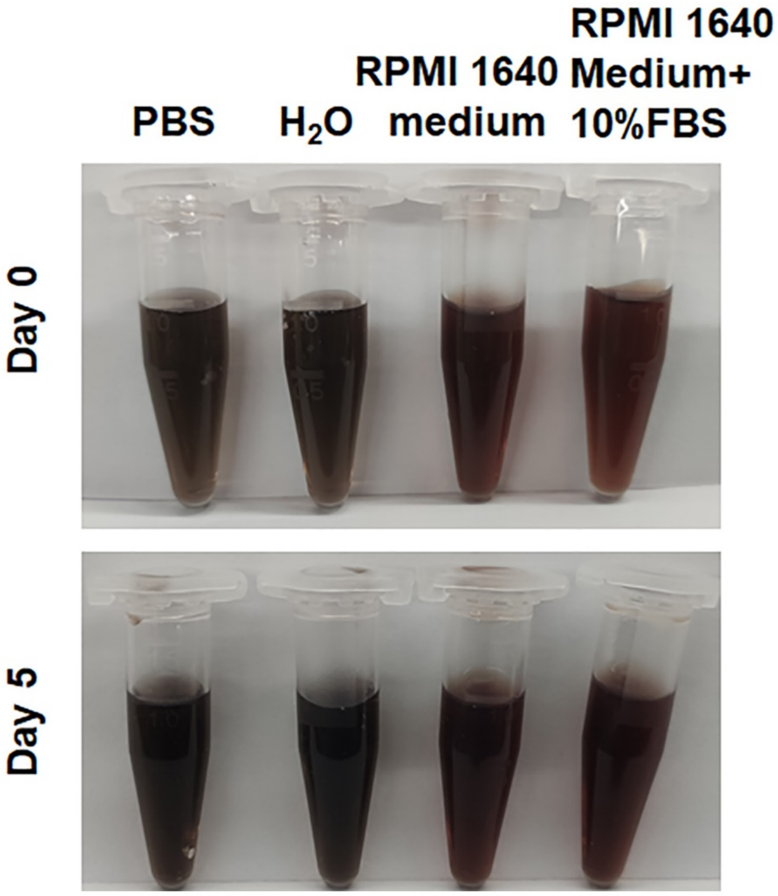

Figure S15. Photos of the AuNR@PEG/PolyRu Ves in H_2O, PBS, RPMI 1640 medium, and RPMI 1640 medium containing 10% fetal bovine serum (FBS). No obvious aggregation was observed after incubation 5 days, indicating the excellent stability of the AuNR@PEG/PolyRu Ves in different physiological solutions.

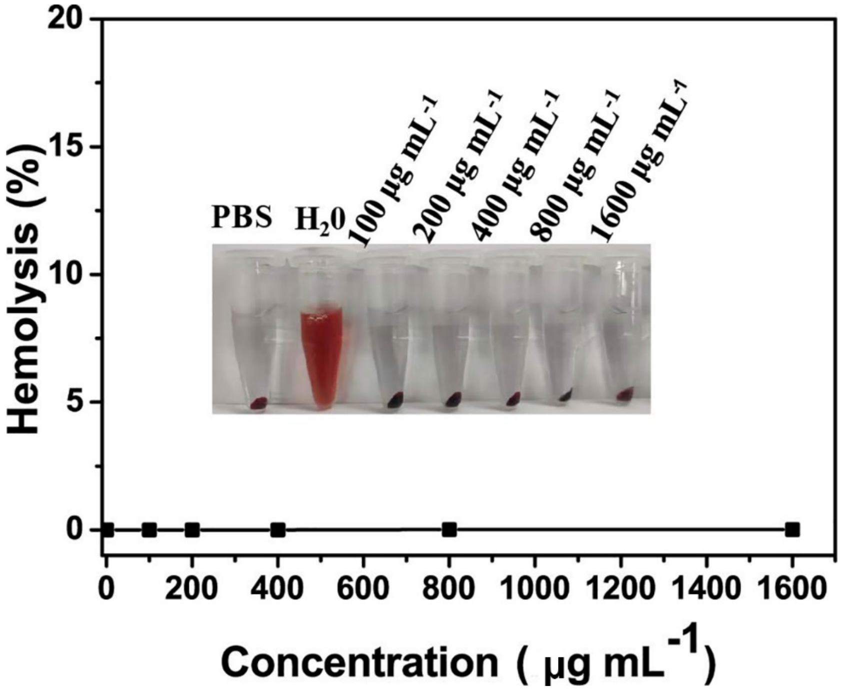

Figure S16. Blood hemolysis using the AuNR@PEG/PolyRu Ves at different concentrations from 100 to 1600 μg mL^{-1}. Water was used as a positive control and PBS was used as a negative control. No visible hemolytic effect was observed when red blood cells were incubated with the nanoparticles for 12 h. Thus, the hybrid NPs were compatible with red blood cells.

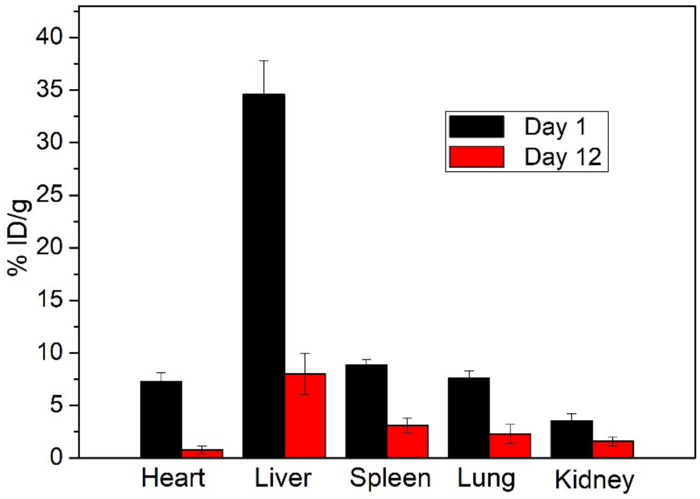

Figure S17. Biodistribution of theVes in major organs at day 1 and day 12 post-injection.

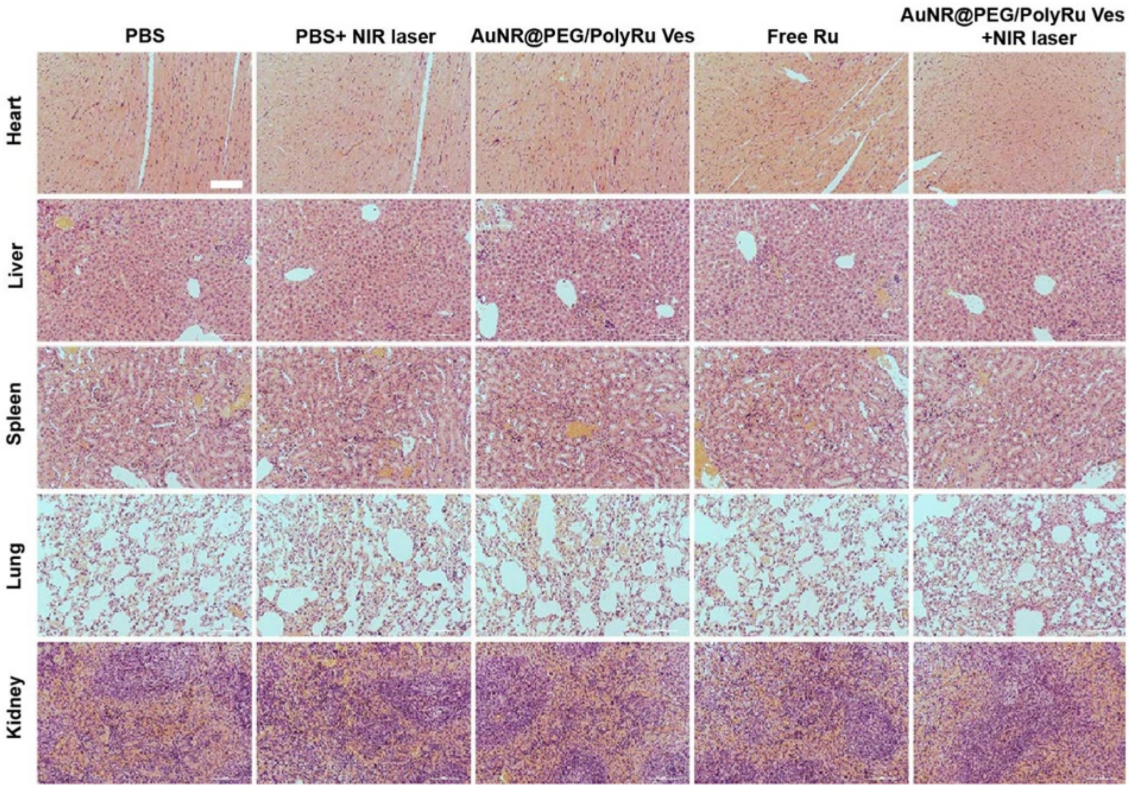

Figure S18. H&E staining of different organ sections (heart, liver, kidney, lung and spleen) obtained from different groups after treatment at 16 days. Scale bars: 100 μm.

References

[S1]V. Bukhanko, P. G. Lacroix, I. Sasaki, M. Tassé, S. Mallet-Ladeira, Z. Voitenko, I. Malfant, *Inorganica Chimica Acta* **2018**, *482*, 195-205.

[S2]W. Sun, M. Parowatkin, W. Steffen, H. J. Butt, V. Mailander, S. Wu, *Adv. Health. Mater.* **2016**, *5*, 467-473.
